# Forest growth in Europe shows diverging large regional trends

**DOI:** 10.1038/s41598-023-41077-6

**Published:** 2023-09-16

**Authors:** Hans Pretzsch, Miren del Río, Catia Arcangeli, Kamil Bielak, Malgorzata Dudzinska, David Ian Forrester, Joachim Klädtke, Ulrich Kohnle, Thomas Ledermann, Robert Matthews, Jürgen Nagel, Ralf Nagel, François Ningre, Thomas Nord-Larsen, Peter Biber

**Affiliations:** 1 Chair of Forest Growth and Yield Science, School of Life Sciences Weihenstephan, Technical University of Munich, Hans-Carl-Von-Carlowitz-Platz 2, 85354 Freising, Germany; 2 Sustainable Forest Management Research Institute iuFOR, University Valladolid, Valladolid, Spain; 3 ICIFOR-INIA, CSIC, Ctra a Coruña km 7.5, 28040 Madrid, Spain; 4 Forest Research, Alice Holt Lodge, Farnham, Surrey UK; 5 Department of Silviculture, Institute of Forest Sciences, Warsaw University of Life Sciences, Warsaw, Poland; 6 Department of Forest Management, Forest Research Institute, Sekocin Stary, Poland; 7 CSIRO Environment, Canberra, ACT 2601 Australia; 8 Swiss Federal Research Institute WSL, Birmensdorf, Switzerland; 9 Forstliche Versuchs- und Forschungsanstalt Baden-Württemberg (FVA), Abteilung Waldwachstum, Freiburg, Germany; 10 Bundesforschungs- und Ausbildungszentrum für Wald, Naturgefahren und Landschaft, Vienna, Austria; 11 Nordwestdeutsche Forstliche Versuchsanstalt Sachgebiet Ertragskunde, Göttingen, Germany; 12 Université de Lorraine, AgroParisTech, INRAE, SILVA, 54000 Nancy, France; 13 Section for Forest and Bioresources, Department of Geosciences and Natural Resource Management, University of Copenhagen, Copenhagen, Denmark

**Keywords:** Forestry, Environmental impact

## Abstract

Forests cover about one-third of Europe’s surface and their growth is essential for climate protection through carbon sequestration and many other economic, environmental, and sociocultural ecosystem services. However, reports on how climate change affects forest growth are contradictory, even for same regions. We used 415 unique long-term experiments including 642 plots across Europe covering seven tree species and surveys from 1878 to 2016, and showed that on average forest growth strongly accelerated since the earliest surveys. Based on a subset of 189 plots in Scots pine (the most widespread tree species in Europe) and high-resolution climate data, we identified clear large-regional differences; growth is strongly increasing in Northern Europe and decreasing in the Southwest. A less pronounced increase, which is probably not mainly driven by climate, prevails on large areas of Western, Central and Eastern Europe. The identified regional growth trends suggest adaptive management on regional level for achieving climate-smart forests.

## Introduction

Effects of environmental changes on forest ecosystems attract attention in more extreme latitudes and altitudes where species lose or gain area at the edge of their ranges^1–3^. At higher boreal latitudes^4–8^ and higher altitudes^9–12^ species may increase growth and vitality, and gain new territories, whereas they may lose at the Mediterranean or lower dry and warm locations^13,14^. However, regional growth trends are less clear across vast areas in the temperate lowlands where productivity is generally higher. While recent studies mostly report increasing growth trends for European trees^15^ and forests^16–18^, the prevailing higher growth, however, is increasingly interrupted by severe drought events^14,19–24^, although growth rates are still larger than historic levels^25,26^. Despite this general pattern, regional reports on trends of tree growth are often contrasting and contradictory, varying between accelerating and decelerating forest growth, even within the same region^27^. Thus, although forest growth and stand productivity are relevant for, among others, wood market, carbon balance, protection function, and habitats^28^, our understanding of long-term trends in different regions is still limited. This inhibits informed decision-making. We use a unique Europe-wide compilation of long-term research plots to (i) quantify the overall growth trend of forest stands, and (ii) break it down into different regions where climate data suggested a spectrum of responses from strongly increasing to declining forest growth.

The study was based on 415 long-term experiments comprising 642 unthinned or only slightly thinned monospecific stands across Europe; the oldest have been surveyed since the 1870’s to the present day (see more information in Supplementary Tables 1–4). The great advantage of these plots is their gapless long-term documentation and well-defined treatment which allows to tell apart natural and silvicultural effects on forest growth. The price to pay for this data quality is, however, that the plots were established independently and thus are not arranged in a perfect spatially representative design. Our data cover seven tree species, most prominently Norway spruce (*Picea abies* (L.) H. Karst.), Scots pine (*Pinus sylvestris* L.), European beech (*Fagus sylvatica* L.), and sessile/common oak (*Quercus petrea* (Mattuschka) Liebl., *Quercus robur* L.). Also included, but less represented were Douglas fir (*Pseudotsuga menziesii* (Mirbel) Franco), European larch (*Larix decidua* Mill.), and silver fir (*Abies alba* Mill.).

The full set of plots, including all seven species, was used to identify the general forest growth trend in Europe, aware of the limited spatial representativity. In a second step, we searched for large-regional trends that could be possibly veiled by the general trend. In order to obtain hints for zones with different growth trends, we used spatially representative climate time series and calculated a climate-vegetation productivity index based on these. An analysis of these data suggested four zones where forest growth should show different trends if climate were its main driver. In our forest plot data, Scots pine, represented with 189 plots, was the geographically most widespread species. Thus, we used these plots for zone-wise growth trend analyses which enabled a regional differentiation and consolidation of information about forest growth in Europe that previously appeared contradictory.

Throughout the study, we expressed stand growth in terms of above-ground woody biomass (i.e. the dry weight of stems and branches, converted from the originally provided wood volume using generalized mass functions^29^, see Supplementary Information 1). We based the study on biomass growth per unit area as it is more comparable across different tree species than volume growth^30^, due to species specific wood densities. Our key goal variable was the periodic annual biomass increment, PAI, which results from dividing the total increment observed between two subsequent surveys of a plot by the time span (in years) between these surveys. As our base model for expressing the relationship between stand age and PAI in our statistical analyses, we used the Hugershoff increment equation^31^, which lends itself nicely to linear mixed modelling approaches. Using mixed models was important to deal with the autocorrelation coming with the nested structure of our data. For fitting species-overarching models, we introduced additional random effects that allowed for species specific curve shapes (see “Methods” section for details).

In contrast to dendrochronologicial studies or the monitoring of growth and mortality of individual trees, the stand level evaluations in this study integrate tree growth, reaction to stress events, and mortality. We harness, for the first time in a formal overarching analysis, a European-wide network of long-term experimental plots, which was originally established for studying long effects of different silvicultural treatment on the stand growth, for a comprehensive growth trend analysis. Here, we use the unthinned and only slightly thinned plots to obtain a consolidated view of different stand growth patterns in Europe and their dependency on climate change. The experimental plots we used were under permanent survey since up to 150 years, and will be under survey in the future, providing a differentiated view on the site specific reaction patterns of forest stands under climate change which is essential for improved understanding and management.

## Results

### General growth trend

**Figure 1:**
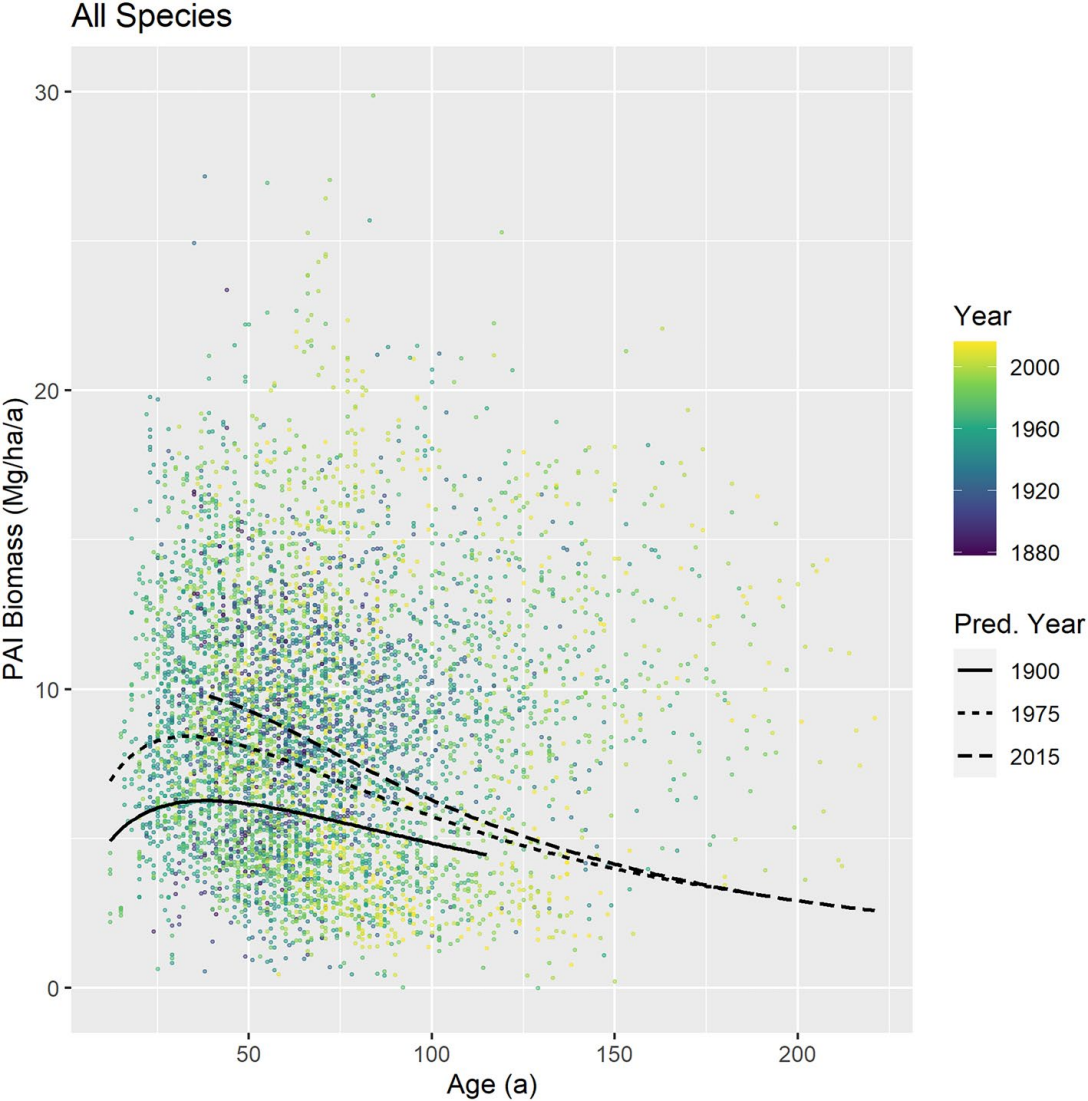
Overall growth trend across all species covered by our data (5815 observations from 642 plots from 415 experiments). Lines: Model predictions for the calendar years 1900, 1975, and 2015, based on the fixed effects of the fitted Eq. (7) (see Supplementary Table 5). The age coverage of the model prediction was cut according to the range of stand establishment years covered by the data. Note that, for the sake of clarity, the diagram’s vertical axis was cut at 30 Mg/ha/a which omits six observations reaching up to 40.7 Mg/ha/a.

The general species-overarching growth trend for the last century, as obtained from a fitted Hugershoff-based model, is shown in Fig. 1 where we compare the expected biomass increment, PAI, over stand age for the calendar years 1900, 1975, and 2015. Note that the model lines in Fig. 1 are not to be read as age-timelines of a single stand. This would only be the case under constant environmental conditions. In reality, however, each line indicates the expected biomass increment of a stand with a given age in the given calendar year. Despite a high variation in the data, the model shows a plausible age trend for constant environmental conditions, i.e. maximum growth at young ages and gradually declining growth with increasing age. More importantly, however, the model reveals a strong increase in PAI with the calendar year, most pronounced at younger stand ages. In other words, a stand that had an age of 40 years in 2015 would be expected to grow about 1.5 times more than a 40-year-old stand in 1900.

For a more quantitative overview, we compared the expected growth for the calendar years 1915 and 2015 for two stand ages, 40 and 90 years (Table 1). These ages represent forest stands at about their maximum growth, and mature stands near to usual final harvest ages. We included not only the above species-overarching model, but also analogue models fitted separately for the four main species covered by our data, i.e. Norway spruce, Scots pine, European beech, and sessile/common oak (Supplementary Fig. 2, Supplementary Tables 6–9). From 1915 to 2015 the annual biomass growth (without species distinction) increased by about 47% in 40-year-old stands and by 29% in 90-year-old stands. In terms of absolute mass growth, this is equivalent to an increase of 3.1 Mg ha^–1^ yr^–1^ in younger and 1.5 Mg ha^–1^ yr^–1^ in mature stands in the last century (Table 1). Remarkably, lower relative increment gains were found for the conifer species Norway spruce and Scots pine (18 and 35%) compared to the deciduous species European beech and sessile/common oak (75 and 46%).

**Table 1:** Species-specific and species-overarching trends of the mean annual mass biomass growth of 40 and 90 years old stands from 1915 to 2015 in terms of absolute and relative changes*.

Species	Biomass growth				Increase from 1915 to 2015			
	1915		2015		Absolute		Relative	
	Mg ha^–1^ a^–1^		Mg ha^–1^ a^–1^		Mg ha^–1^ a^–1^		%	
	40 a	90 a	40 a	90 a	40 a	90 a	40 a	90 a
Norway spruce	8.9	7.5	10.5	8.8	1.6	1.3	18	18
Scots pine	4.1	2.9	5.5	4.0	1.4	1.0	35	35
European beech	8.2	9.0	14.3	15.7	6.1	6.7	75	75
Sessile/common oak	8.2	8.6	11.9	12.5	3.7	3.9	46	46
All species (as in Fig. 1)	6.6	5.3	9.7	6.9	3.1	1.5	47	29

*Values were obtained from the fitted model shown in Fig. 1 (Eq. 7, Supplementary Table 5) and analogue species specific models (Eq. 8, Supplementary Tables 6–9). Numbers do not fully match due to rounding errors.

### Regionalized climate trends

**Figure 2:**
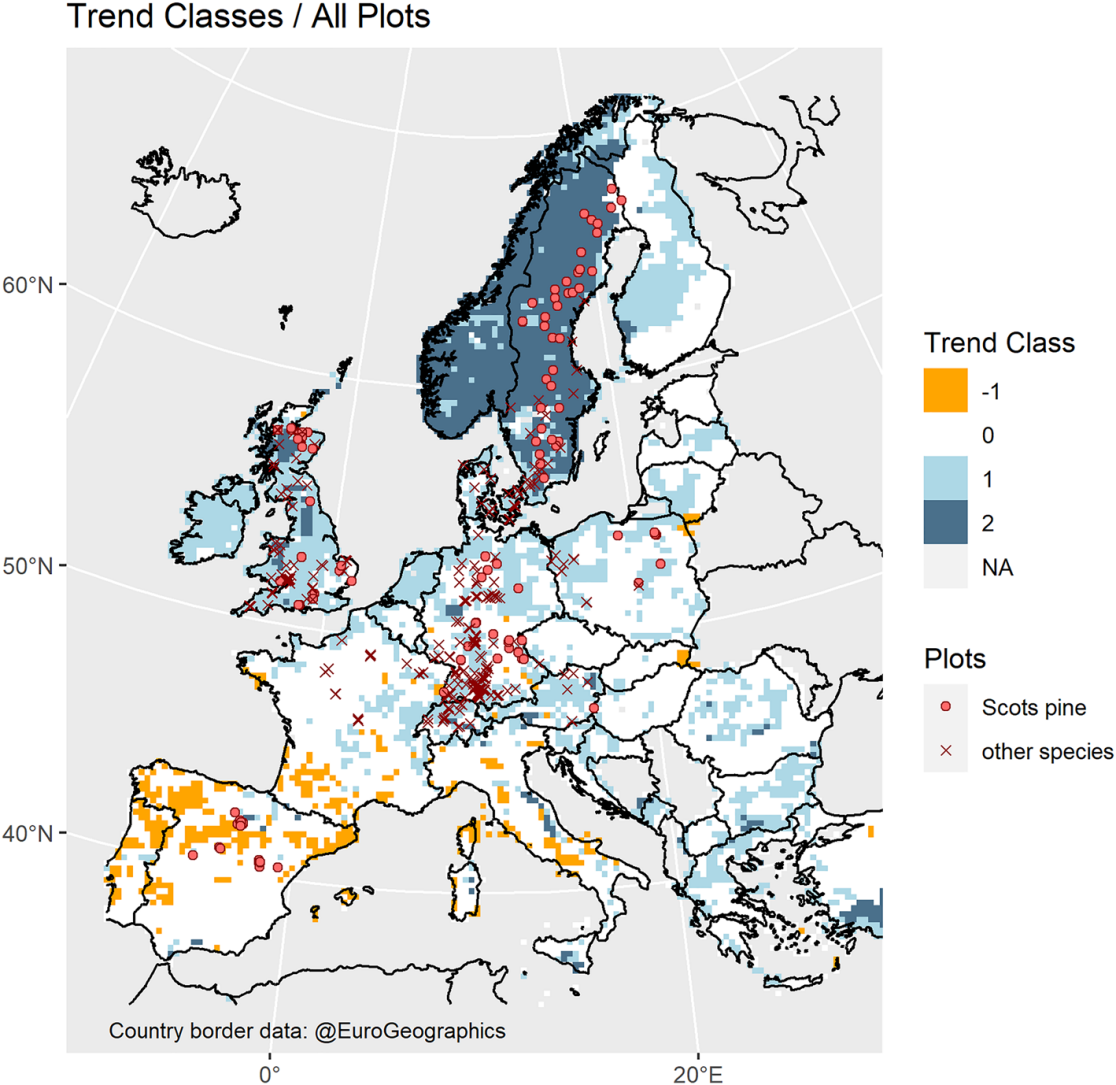
Distribution of plots and climate vegetation productivity trend classes as determined by changes in the CVP index^33^ during the period of 1975–2017. The trend classes − 1, 0, 1, and 2 suggest declining, no clear trend, improving, and strongly improving climate conditions for forest growth, respectively. The number of plots is 642 in total, and 189 for Scots pine; note, that the symbols of plots often mask each other due to their close vicinity and the scale of the map. See Supplementary Fig. 1 for the continuous trend values before classification.

As a framework for identifying regional trends in our forest growth data, we analyzed gridded spatial climate data obtained from the JRC MARS Meteorological Database, which were available at a 25 km spatial resolution and daily temporal resolution from 1975 to 2017^32^. Our findings suggested four different classes of potential climate-induced forest growth trends, whereby each gridpoint was attributed to one class (Fig. 2). This classification was achieved by testing the temporal change of the annual climate vegetation productivity index CVP by Paterson^33^ for significance and strength on each gridpoint. The largest areas in Europe are covered by the classes 0 and 1 which suggested no clear trend and a significant increase in forest growth, respectively. A spatial mix of these two classes prevails from Central to Eastern Europe, extending from Finland to Greece in a North–South direction. In both zones, conditions have been favourable for tree growth during the entire time span of the analysis, but in class 1, warming still improved conditions to some degree. In general, class 1 areas were found along the west facing coastline in England, the Netherlands, and Finland, where warmer climate was accompanied by ample rainfall. Virtually the whole area of Norway and Sweden, but also parts of Scotland, was included in class 2 for which our analyses indicated a large improvement in forest growth conditions. Here, the limits set to forest growth by low temperatures and short growth season, seemed to have weakened throughout the time covered by the data. In parts of Southern and Southwestern Europe, we found, in combination with an indifferent trend, locations where the climate conditions for forest growth deteriorated during the last four decades. This was mainly the case in the northern half of the Iberian Peninsula, but also in southwestern France and the western parts of Italy. In these parts, heat seemed to be increasingly limiting tree growth due to water shortage during parts of the growing season.

### Regionalized growth trends

**Figure 3:**
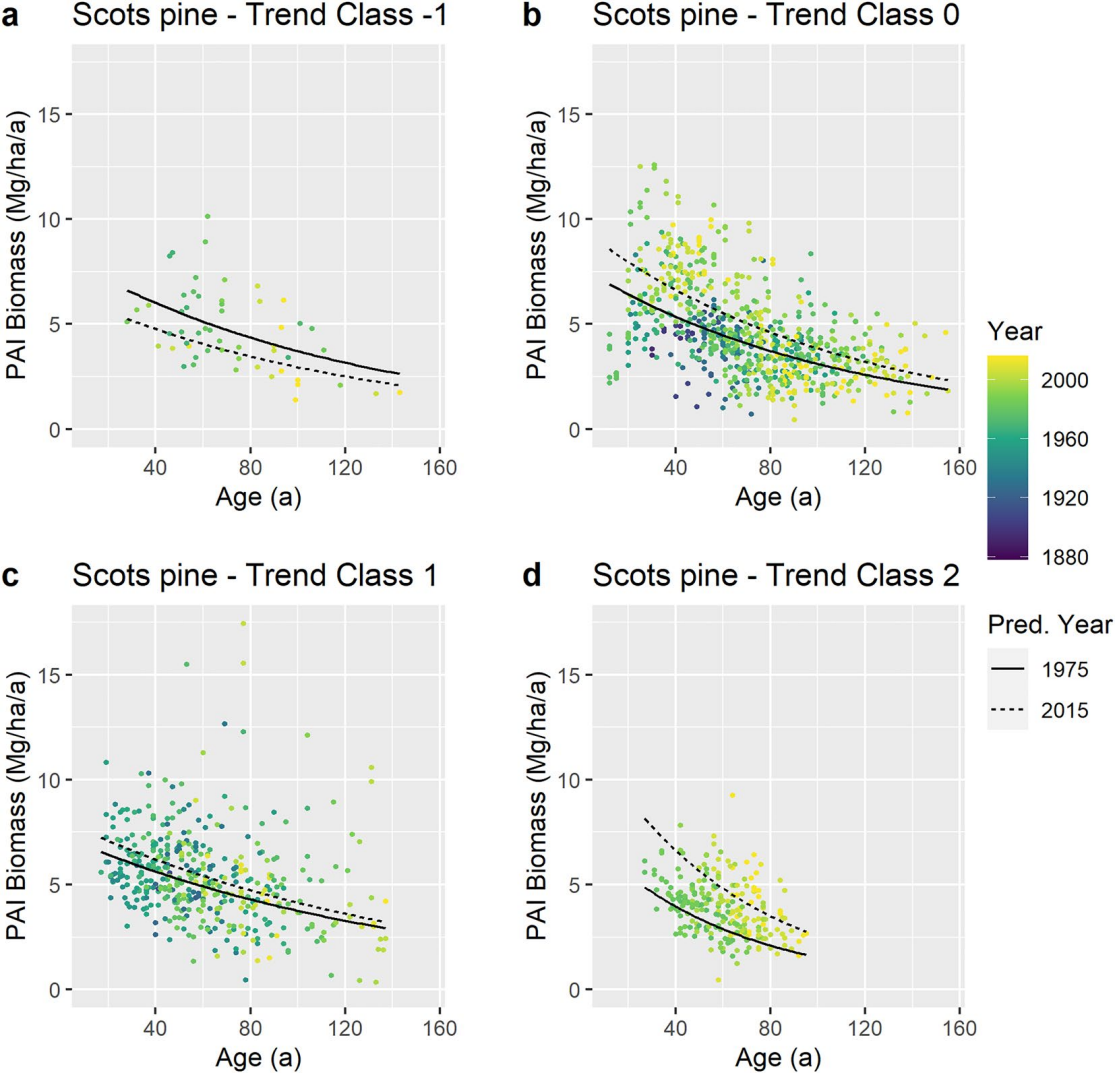
Visualisation of class-wise growth trends for Scots pine. Lines: Model predictions for the calendar years 1975 and 2015 (period of climate data coverage). The predictions based on the fixed effects of the fitted Eq. (9) (see Supplementary Table 10). Data in diagrams (**a**) trend class −1, 55 observations from 8 plots, (**b**) trend class 0, 734 observations from 87 plots, (**c**) trend class 1, 436 observations from 50 plots, (**d**) trend class 2, 228 observations from 44 plots.

While our spatially and species overarching analysis above revealed a general trend of increasing growth, we tested whether the growth trend zonation suggested by the climate data was confirmed by the growth trends observed on the long-term research plots. To this end, we focused on the Scots pine plots, which by far had the widest spatial coverage, including trend class −1, where the climate trend suggested decreasing growth. Based on an extended version of the regression model used for identifying the overall trends, we were able to show different trends dependent on which trend class a plot was attributed to (Fig. 3).

Most notable, for both extremes, the classes − 1 and 2, where the CVP index suggested a decline, and a strong increase in growth, respectively, this was clearly confirmed by the Scots pine plots. In line with the the CVP trend, we also observed a positive but weaker growth trend for Scots pine growing in class 1 areas. We found, however, a significant increase in Scots pine growth in the climate trend class 0, where the climate data did not indicate a clear growth trend. Our interpretation is that in the extreme classes −1 and 2 climate indeed sets the limits for forest growth and is, therefore, the main cause of the observed growth trends. In classes 0 and 1, where the climate conditions have been in the comfort zone of our main tree species during the whole time of observation, other factors, like, prominently, nitrogen deposition might overlay the climate effects^18,34,35^.

Given these regionalized growth trends of Scots pine, we attempted a rough projection to the European level (Table 2). To this end, we used the areas covered by our climate analysis as shown in Fig. 2 (i.e. the EU states, Norway, Great Britain, and the western edge of Turkey). Based on recent tree species coverage maps^36^, it was possible to estimate the areas covered by Scots pine stands in each trend class throughout the whole area of interest. Combining this area information with the fitted model shown in Fig. 3 we estimated the zone-wise biomass growth of 75-year-old Scots pine stands for the years 1975 and 2015 (which frame the time span covered by our climate data). The age of 75 was chosen as this is about the age where the periodic annual increment, PAI (which our model estimates), of Scots pine stands equals the mean annual increment, MAI. Therefore, we considered the PAI at age 75 a reasonable estimate of the average growth of all Scots pine stands in each zone.

**Table 2:** Upscaling of the expected biomass growth trends for Scots pine to the European level*.

Trend class	Area covered by Scots pine	Biomass growth		Change from 1975 to 2015	
		1975	2015	Absolute	Relative
	km^2^	Gg a^–1^	Gg a^–1^	Gg a^–1^	%
− 1	8990	4081	3238	− 843	− 21
0	205,098	79,963	99,069	19,106	24
1	134,263	59,632	65,727	6095	10
2	142,181	32,246	53,969	21,723	67
Total	490,532	175,922	222,003	46,080	26

*The growth estimates come from the model shown in Fig. 3 (based on the fixed effects of the fitted Eq. (9) (see Supplementary Table 10), whereby a stand age of 75 years has been assumed; the time frame is 1975 to 2015. For the locations of the trend classes −1 to 2 see Fig. 2; the areas reported here correspond exactly to those shown in Fig. 2. Species area coverages were calculated based on recent tree species coverage maps^36^.

The total area covered by Scots pine in the domain of interest currently amounts to about 491,000 km^2^, and our fitted model (as visualized in Fig. 3) suggested an increased above-ground woody biomass growth of 46,000 Gg/a (i.e. 26%) compared to 1975. Most remarkable is the estimated production increase in trend class 2 (mostly Sweden and Norway) where on an area of about 142,000 km^2^ we estimate a production increase of 22,000 Gg/a which means a plus of 67% in relation to 1975. Almost the same absolute increase, but only 24% in relative terms, is estimated for the considerably larger zone 0, where apparently climate change is not the main driver of the growth acceleration. For the somewhat smaller trend class 1, where climate change suggests a positive growth trend, the estimated relative growth acceleration amounts to 10% only. While the relative growth deceleration in class −1 amounts to 21%, the area covered by this class is relatively small, i.e. 2% of the whole area of Scots pine, which makes a minor absolute contribution (− 843 Gg/a) to the overall balance. Note that we took the areas covered by Scots pine as constant over time for the projections. Even though tree species areas certainly changed over the four decades, this is a relatively slow process, and it does not impair the main message that the areas where we observed dissimilar growth trends are quite different and that this fact is important for the overall balance.

## Discussion

Many studies on the current or future growth of forests are based on regional case studies^37,38^, simulation models^39,40^, or inventories spanning only a few decades^17,27,41^. There are important disadvantages and limitations of such approaches. Case studies show patterns that often contrast between regions, e.g. for European beech^13,18,42,43^. Modern remote sensing or grid based inventory data are often strongly limited in the length of times series and the growth of the surveyed stands may be confounded by forest management effects^16,17^. Compared to those sources, records from long-term experiments provide invaluable information and may contribute to a more consolidated view on forest growth conditions in Europe. Compared with other data, fully stocked and unthinned or slightly thinned experimental plots used in this study were not impaired by thinnings of unknown strength and intensity but represent changes of the actual growth potential.

Having mentioned the advantages of long-term plot data, we must also point out their prominent disadvantage, which is the missing overall spatial design that leads to an incomplete coverage and uneven coverage intensities, with large gaps especially in Eastern and Southern Europe. Insofar, while our quantiative results are trustworthy as such, the precision when we upscale our findings must be considered low. This might be slightly more the case for the general growth trend and less for the zone-wise trends. This is because the zone wise trends are coupled to spatially representative information that links an important driver – climate - to forest growth. The connection of the two data sources was partly driven by the idea of balancing the jagged spatial representativeness of the growth data to some extent. Thus, we assume, our approach makes good use of the advantages of long-term plots while trying to minimize their main disadvantage.

As we base our study on stand level findings over long time and across wide parts of Europe our study has the potential to reconcile so far diverging findings. Stand level growth data include mortality at the tree level, growth reactions on stress events, and growth decline of individual trees. Most dendrochronological or dendroecological studies do not scale up to absolute growth rates at the stand level^21,44^. Studies of individual tree growth or mortality hardly consider that surviving trees can partly buffer growth losses and mortality^45^. Studies focused on individual drought events often neglect that tree and stand growth can partly compensate growth losses in post drought phases^46,47^.

The average increase of biomass growth by 29–47% across Europe in the last 100 years (Table 1) is in line with many previous regional studies^18,25,48^. Extended growing seasons and increases in atmospheric CO_2_-concentrations^49^ are considered to be the main causes of this growth trend, especially when combined with N-deposition^50–52^, which steadily increased eutrophication especially in central and northern Europe^53^. Given that we only included fully stocked stands that were lightly thinned at most, silvicultural treatments are unlikely to have contributed to the growth responses. Nutrient exports due to the widely applied practice of litter raking^54,55^ could potentially have influenced the results; however, this was gradually discontinued by the middle of the last century and was typically stopped when plots were established. Tree breeding, especially common in Scandinavia, may have increased volume growth by 10 to 25%^56,57^. This could have contributed to the increased growth seen in Scandinavia but is less likely to have had an influence in other regions. We are not aware of any correlations between the time of plot establishment and quality of the selected sites that could have counfounded our results. In addition, the long-term time series represented by our plots and our choice of random effects in our statistical models should make our approach robust in this regard.

With the data from our Scots pine plots, we were at least partly able to disentangle the conglomerate of reported large-regional growth trends and their causes. Scots pine was the geographically most widespread species in our data, and covered all of the four trend classes identified with the climate data. Therefore, among the species covered by our data, Scots pine was most suitable for bioindication. We found the strongest growth acceleration in the boreal zone (trend class 2), namely in Norway and Sweden, with initially unfavourable but strongly improving climate conditions for forest growth (measured in log(CVP)). Here, any growth limitations by temperature and length of the growing season in the past seem to have greatly weakened due to global warming, coming with increasing N-depositions in addition^53^.

The growth decline in parts of the Mediterranean region (trend class −1) suggests that reductions in precipitation together with rising temperatures are increasingly limiting factors for growth^58^. No such clear picture was obtained for the trend classes 0 and 1 which cover greater parts of Western Europe, the British Isles, Central and Eastern Europe. Here, in contrast to trend classes −1 and 2, climate conditions have neither in the distant nor in the recent past been limiting forest growth. Previous limitations set by nutrient supply seem to weaken due to N-depositions and increased CO_2_ concentrations which leads to a considerable, but comparably moderate increase in forest growth throughout the large region.

In summary, our results suggest the existence of three large growth trend regions in Europe. For two of them we were able to establish a predominant climate effect while in the third, other factors than climate seem to be the prevalent driving forces. More precisely, in the western boreal region (Norway, Sweden, trend class 2), previous limits imposed by climate have been strongly weakening up to the present day, triggering a vigorous growth response. The contrary is true in parts of the Mediterannean zone (trend class −1) where we observe declining forest growth due to strengthening climatic limits. In the third zone, where a mix of trend classes 0 and 1 prevails, climate seems not to be the main factor determining the observed trends of a moderately increasing forest growth.

A recently published study^59^ reports correlations of increasing atmospheric CO2 with the nutritional status of forest trees in Europe. Interestingly, in Northern Europe, the improving climatic conditions seem to come with increases of foliar nutrient concentrations, including N. For Mediterranean forests, in contrast, a declining nutritional status is reported. Both effects might reinforcingly contribute to the trends we identified in the boreal region and parts of the Mediterranean region. A declining nutritional status was also reported for temperate forests^59^, however, it was not reflected in the growth trends we found with trend classes 0 and 1. This might be taken as a signal that the increasing trend we found mainly for Central and Eastern Europe might not be stable in the future.

Due to the management applied on our plots, their stand density was never reduced below a level that would impair their growth potential. This important requirement could be met because permanent research plots are documented without gaps. We were thus able to exclude plots that were not fully stocked at any time during their observation from the outset. Insofar, forest practice could profit from comparing the increments measured in their ongoing inventories with our estimates for the zone of interest. Strong deviations would indicate that practitioners keep the stands at densities which are below the current optimum increment. This may be desired for other reasons (e.g. stand stability, elite-tree concepts), but should always result from an informed decision. For the parts of the Mediterranean region where our results suggest a deceleration of the growth potential, a critical scrutiny of the current felling plans is recommended in order to avoid overharvesting and an excessive increment reduction.

We compiled a comprehensive dataset from long-term forest experiments, the first of which were established in the late nineteenth century, and arrived at a regionally differentiated overview of growth trends in Europe. This allowed a consolidation of reports of increasing versus decreasing forest growth trends in Europe that previously seemed contradictory. Large-scale regional changes in growth rates will possibly modify the flows of commodities including round wood and wood products from regions of higher to lower productivity. For the whole area, our results still suggest an increasing potential for carbon sequestration, however coming with a considerable regional shift with accelerating growth in some regions and decelerating growth in others. While the reported spatial and temporal growth trends are essential for the quantity of sustainable wood production, most other forest ecosystem functions and services, such as nutrient and water cycling, carbon assimilation and storage, or many protection functions are closely linked to the level and age related development of growth^60,61^. Thus, socio-economic consequences could be expected e.g. an amplification of the south-north gradient of employment and capital in the forest and wood sector.

In the public, a Black and White thinking about the health state of the forest is very common; some see all forests dying, others see no problems with the forest at all. Regarding active forest management, the variety of opinions is similar; some people want all forests protected, others vote for comprehensive forest management. Our results show that thriving of the forest here and declining there is no contradiction but a question of site conditions. We provide a differentiated view of the state of the forest, which is important for both, understanding the effects of climate change and appropriate management measures for mitigation and adaption.

## Methods

### Data

This study is based on two different data sets, forest growth data from long-term plots, and grid based climate data. Both will be dealt with in more detail below, but it is important to state before, that the forest growth data provide invaluable time series on the one hand, while on the other hand, their spatial representativeness is limited. By connecting the research plot data with spatially representative climate data, we intended to compensate for their patchy spatial representativeness to a certain extent.

The 642 long-term plots included in this study are located in nine countries across Europe and were provided by eleven institutions (see Supplementary Table 1). They cover a broad variety of soil and climate conditions. The extremes covered by our climate data were annual precipitation sums of 422–2333 mm, and − 0.3–12.8 °C mean annual temperature (see Supplementary Table 2); they represent Mediterranean, Atlantic, continental, as well as boreal ecoregions in Europe (see Supplementary Table 3). The establishment and survey of these long-term experiments in Central European forests started with the foundation of the Association of German Forest Research Stations in 1872^62^, and was extended to Europe and later worldwide by the International Union of Forest Research Organization founded in 1892^63,64^. The long-term experiments were established, measured, silviculturally steered, and evaluated as set out by the abovementioned organizations^65–68^.

### Calculating growth and yield characteristics at the stand level

The raw data of each survey comprised the plot size, the stem diameter of all trees at 1.3 m height, information about whether the tree was removed or remained in the stand, and the tree heights measured on all trees or on a sample of 30–50 trees covering the whole stem diameter range. The inventories were repeated every 3–12 years and up to 31 times. Typical plot sizes are between 2000 and 5000 m^2^. Proven standard procedures were available^69^ for calculating stand sum values per ha (most importantly wood volume and its increment) and mean values (e.g. quadratic mean stem diameter and corresponding height). For the stand level stem volume, this was done in three steps. First, each survey’s height measurement sample was used to fit a dbh-height curve which allowed to estimate the missing tree heights. Second, regional species specific volume functions were applied for calculating each tree’s stem volume with dbh and height as input variables. Third, the single tree volumes were added together in order to obtain the whole stand’s stem volume, and scaled up to 1 ha. While the individual height measurement errors certainly affect the volume estimates on single tree level, they practically cancel out each other in the stand level volume. Up to that step, the calculations were individually carried out at the institutions that are managing the research plots as part of their standard data evaluation protocols. Thus, the most appropriate regional volume functions and height curve equations were used in each single case.The subsequent steps were conducted centrally with the same functions for all plots: The stand level stem volumes were upscaled to aboveground woody volume based on generalized allometric functions^29^, and converted to biomass with species-specific wood density values^70^ (see Supplementary Information 1 for details). The periodic annual stand level biomass increment between two subsequent surveys, PAI, the goal variable of our statistical models, was calculated as follows:

1 $${\text{PAI}}=\frac{{M}_{2}-{M}_{1}+{M}_{r}}{{t}_{2}-{t}_{1}}.$$

With ${t}_{1}$ and ${t}_{2}$ being the calendar years or stand ages at two subsequent surveys, and ${M}_{1}$ and ${M}_{2}$ being the remaining biomass at surveys 1 and 2, respectively. ${M}_{r}$ is the biomass of the trees that were removed or died after survey 1, including trees which were removed exactly at ${t}_{2}$. We calculated, in addition, the total biomass yield, TY, which is, at a given point in time, the standing biomass at that time plus the total biomass that has been removed or died up to that time. When TY is known, dividing it by the corresponding stand age yields the mean annual increment, MAI, whose maximum indicates the rotation time of maximum production.

See Supplementary Tables 4 and 11 for an overview of the plots’ most important stand level characteristics.

### Spatio-temporal climate-vegetation-productivity index values

The EU’s JRC MARS Meteorological Database makes Europe wide climate data available at a spatial resolution of 25 × 25 km^2^ and in daily temporal resolution^32^. At the time of access (July 2018) these data covered the years from 1975 to 2017. This enabled us to calculate annual values of Paterson’s Climate-Vegetation-Productivity Index CVP^33^ for each single gridpoint. The CVP is a theory-based approach that has been used for mapping the world’s forests’ productivity as early as in the 1950’s^33^. Recently, the CVP came back to attention in the context of climate change and forest productivity^71,72^. The CVP is calculated as

2 $${\text{CVP}}=\frac{{T}_{v}\cdot P\cdot G\cdot E}{{T}_{a}\cdot 12\cdot 100},$$

with the maximum mean monthly temperature, ${T}_{v}$ (°C), the difference between the warmest and the coldest monthly temperature mean, ${T}_{a}$ (°C), the annual precipitation, $P$ (mm), the length of the growing period in months, $G$, and the so-called evapotranspiration reducer or radiation ratio, *E*., i.e. the annual radiation at the pole related to the annual radiation at the latitude of interest in percent. The length of the vegetation period $G$ can be limited by cold temperatures or by arid conditions; following the standard of climate diagrams, we took *G* as the number of months, whose mean temperature was at least 5 °C, and where the monthly mean temperature multiplied by 2 was smaller than the monthly precipitation in mm^73^. In this way, also relevant changes in annual precipitation and temperature patterns are taken into account, because only those months are counted where both, precipitation and temperature allow for forest growth.

For estimating the evapotranspiration reducer $E$ dependent on the geographical latitude, Paterson provided a graphical function^33^ which was digitized for automatized application in this study. As Paterson showed that forest productivity in m^3^ ha^–1^ yr^–1^ could be estimated as a linear function of $\mathrm{ln}({\text{CVP}})$, we used the logarithmic transformation in this study.

### Pointwise climate-vegetation-productivity trends

In order to identify temporal trends in the development of ln(CVP) from 1975 to 2017, we fitted the following regression model to the data of each gridpoint separately:

3 $$\mathrm{ln}\left({\text{CVP}}_{i}\right)={a}_{0}+{a}_{1}\cdot {\text{year}}_{i}+{\varepsilon }_{i}\,\hspace{2 em}\mathrm{with}\hspace{1 em}{\varepsilon }_{i}=\rho \cdot {\varepsilon }_{i-1}+{u}_{i}.$$

This model describes ln(CVP) as a linear function of the calendar year with the intercept ${a}_{0}$ and the slope ${a}_{1}$. The index *i* sequences the observations in ascending order. The error terms $\varepsilon$ are subject to a first order autocorrelation model with the autocorrelation parameter $\rho$ and i.i.d. errors ${u}_{i}$ with $E\left({u}_{i}\right)=0$. Clearly, this model indicates a temporal trend when ${a}_{1}$ is significantly different from zero, and we used this information for defining the climate trend classes shown in Fig. 2 (see Supplementary Fig. 1 for a map of the slopes before classification). Grid points with a significant slope ${a}_{1}<0$ (i.e. decreasing ln(CVP) over time) were attributed to trend class “− 1” (declining climate conditions for forest growth); points with non-significant slopes were attributed to zone “0” (no trend in climate conditions). For significant ${a}_{1}>0$ (i.e. increasing ln(CVP)), their broad range suggested a subdivision into two classes, namely “1” (improvement of climate conditions) and “2” (strong improvement). To that end, we defined a simple threshold. If the slope suggested an increase of ln(CVP) of at least 1.1 over a period of 40 years, it was attributed to trend class 2, and otherwise to class 1. This threshold ($\mathrm{ln}\left({\text{CVP}}\right)=1.1$) is equivalent to a 20% increase related to the overall average of ln(CVP) = 5.5 in 1975. Note that we set the significance requirement for ${a}_{1}$ to $p<0.1$ (instead of the usual 0.05) in order to lower the risk of type II errors (erroneously assuming no trend).

### Basic model for growth trend analysis

The core of all growth trend analyses applied in this study is the Hugershoff equation^31^ (Hugershoff^31^), which can be used for describing the periodic annual stand biomass increment ${\text{PAI}}$ as a function of the stand age ${\text{AGE}}$:

4 $${\text{PAI}}=b\cdot {\text{AGE}}^{{a}_{1}}\cdot {\text{e}}^{{a}_{2}\cdot {\text{AGE}}}.$$

Here, $b$ is a positive scaling parameter, and as long as the parameters ${a}_{1}$ and ${a}_{2}$ have different signs, this equation describes a typical increment pattern over time; with accelerating growth at an early stage, a subsequent phase of maximum growth, which is followed by a phase of receding increment. For statistical modeling, the Hugershoff equation is especially useful as it can be linearized by taking its natural logarithm:

5 $${\mathrm{ln}\left({\text{PAI}}\right)=a}_{0}+{a}_{1}\cdot \mathrm{ln}\left({\text{AGE}}\right)+{a}_{2}\cdot \text{AGE }\hspace{2 em}\text{with }\hspace{1 em}{a}_{0}=\mathrm{ln}\left(b\right).$$

This linear form allowed us to fit an actual non-linear biologically plausible equation inside the robust framework of linear mixed regression. Moreover, this transformation means that the model’s error structure after backtransformation is multiplicative, and that the fixed effect variables we added in the extended models below (Eqs. 6, 7, 8, 9) actually have a multiplicative effect on growth. Both effects are desired due to the inherently multiplicative error structure of biological growth processes^74^.

In order to include potential growth trends, we introduced the calendar year when the stand was established, EYEAR, as follows:

6 $$\mathrm{ln}\left({\text{PAI}}\right)={a}_{0}+{a}_{1}\cdot \mathrm{ln}\left({\text{AGE}}\right)+{a}_{2}\cdot {\text{AGE}}+{a}_{3}\cdot {\text{EYEAR}}+{a}_{4}\cdot {\text{AGE}}\cdot \text{EYEAR.}$$

The interaction of EYEAR and ln(AGE) was not included, because this never improved the plausibility of the results or the goodness of fit. This basic model was used to describe the periodic annual biomass increment as a function of the stand age and the calendar year when the stand was established. The introduction of EYEAR allowed for a changing level of growth due to growth trends, while the interaction of EYEAR and AGE, also allowed for a change in the pattern of growth. Note, that in all statistical models based on Eq. (6), ${\text{AGE}}$ was always the real stand age divided by 10, and ${\text{EYEAR}}$ was always the actual calendar year divided by 1000. This kept both variables at a similar order of magnitude, which lead to a more stable behaviour of the model fitting algorithm.

### Overarching growth trend analysis

For the statistical analysis of the overall growth trend across all species and without regional trends (results shown in Fig. 1), Eq. (6) was extended to form the following mixed linear model:

7 $$\mathrm{ln}\left({\text{PAI}}_{ijk,s}\right)={a}_{0}+{a}_{1}\cdot \mathrm{ln}\left({\text{AGE}}_{ijk,s}\right)+{a}_{2}\cdot {\text{AGE}}_{ijk,s}+{a}_{3}\cdot {\text{EYEAR}}_{ij,s}+{a}_{4}\cdot {\text{AGE}}_{ijk,s}\cdot {\text{EYEAR}}_{ij,s}+{b}_{i}+{b}_{s}+{c}_{s}\cdot {\text{AGE}}_{ijk,s}+{\varepsilon }_{ijk,s}.$$

In this model the indexes $i$, $j$, and $k$ represented the *i*th trial, the *j*th plot in trial *i*, and the *k*th observation of plot *j* in trial *i*. While these levels were nested, the index $s$, which indicated the tree species, was treated as outside this nesting. The random effects in the model are ${b}_{i}$, ${b}_{s}$, and ${c}_{s}$ (${b}_{i}\sim N\left(0,{\tau }_{1}^{2}\right), {b}_{s}\sim N\left(0,{\tau }_{2}^{2}\right), {c}_{s}\sim N(0,{\tau }_{3}^{2})$). The first random effect, ${b}_{i}$, was connected to the level of the trial and took into account the fact that all observations from the same trial were not statistically independent. As each trial has its own site conditions, and the plots inside a trial are comparable in this respect, this random effect also covers different levels of growth resulting from different site conditions. In preliminary modelling phases, we also included an analogous random effect on the plot level, ${b}_{ij}$, which, however, did not improve the model fits compared to fitting Eq. (7). The random effect ${b}_{s}$ allowed for species specific scaling, and the random effect ${c}_{s}$ enabled species-specific growth patterns (e.g. typically, the age of maximum growth would be lower for Scots pine compared to European beech). Due to these species-specific random effects, the fixed effect parameters ${a}_{0}, {a}_{1}, \dots , {a}_{4}$ expressed the growth and growth trend of “the average species” on “the average site”. The model predictions in Fig. 1 were generated with these fixed effects only, i.e. they describe the expected biomass increment across species at a given age in a given calendar year (which is the year of stand establishment plus the stand age. Finally ${\varepsilon }_{ijk,s}$ represents independently and identically distributed errors (${\varepsilon }_{ijk,s}\sim N(0,{\sigma }^{2})$). See Supplementary Table 5 for the parameter estimates and significances, and Fig. 1 for a visualization of model predictions.

On the same basis, we fitted growth trend models for the four main species in our data, Norway spruce, Scots pine, European beech, and sessile/common oak separately. In no case was the interaction of AGE and EYEAR significant; it was therefore left out of the final species-specific model, which is:

8 $$\mathrm{ln}\left({\text{PAI}}_{ijk}\right)={a}_{0}+{a}_{1}\cdot \mathrm{ln}\left({\text{AGE}}_{ijk}\right)+{a}_{2}\cdot {\text{AGE}}_{ijk}+{a}_{3}\cdot {\text{EYEAR}}_{ij}+{b}_{i}+{b}_{ij}+{\varepsilon }_{ijk}.$$

Naturally, this model does not contain any species-specific random effects (unlike the species-overarching model (Eq. 7)), but note that for all species except Scots pine, including a random effect on plot level, ${b}_{ij}\sim N(0,{\tau }_{4}^{2})$, improved the model fits and was therefore included. In the case of sessile/common oak, the main effect of AGE turned out non-significant (in contrast to ln(AGE)) and was therefore omitted from the model. The absence of multicollinearity among the fixed effect variables was verified for all PAI models presented in this study by visual display and by calculating variance inflation factors. The species-specific biomass growth estimates in Table 1 are based on these models, see also Supplementary Tables 6–9 and Supplementary Fig. 2 for the fit results and further visualization. For fitting this and all models shown below, we used the function *lmer* from the R package *lme4*^75^ in combination with the package *lmerTest*^76^.

### Trend class specific analysis for Scots pine

As Scots pine was the geographically most widespread species in our data and the only species present in all four identified climate trend classes, we focused on this species for when searching for regionally different growth trends. To this end, we formulated a base model starting with Eq. (6) but adding a categorial variable, TCLASS, describing the climate trend class affiliation of each plot (Fig. 2). The trend class for each plot was obtained by mapping the plot location to the nearest gridpoint of the climate data, with a minimum, average, and maximum plot-gridpoint distance of 0.75 km, 9.7 km, and 17.2 km, respectively.

The variable TCLASS was introduced as a main effect and as an interaction with AGE and EYEAR. Other and higher interactions were not considered, because this did not increase model plausibility while considerably increasing complexity. TCLASS was dummy-coded with trend class −1 as the reference, i.e. TCLASS(0), TCLASS(1), and TCLASS(2) have the value 1 if a plot is in trend class 0, 1, and 2, respectively, and the value 0 otherwise.

When fitting the model, we followed a strict procedure^77^, starting with the full model as described above and leaving out non-significant effects, beginning with the most complex model components (interactions). When there were significant interactions where the contributing main effects were not significant, both the interactions and main effects were kept in the model. As the choice of the reference level of a dummy-coded variable is arbitrary and does not change the model’s goodness-of-fit, the decision was always made regarding the significance of the whole variable TCLASS, not its single levels. This procedure yielded the following model (we keep the numeration of the parameters from Eq. (6) in order to facilitate comparison):

9 $$\mathrm{ln}\left({\text{PAI}}_{ijk}\right)={a}_{0}+{a}_{2}\cdot {\text{AGE}}_{ijk}+{a}_{3}\cdot {\text{EYEAR}}_{ij}+{a}_{5}\cdot {\text{TCLASS(0)}}_{ij}+{a}_{6}\cdot {{\text{TCLASS}}\left(1\right)}_{ij}+{a}_{7}\cdot {\text{TCLASS(2)}}_{ij}+{(a}_{8}\cdot {\text{TCLASS(0)}}_{ij}+{a}_{9}\cdot {\text{TCLASS(1)}}_{ij}+{a}_{10}\cdot {\text{TCLASS(2)}}_{ij})\cdot {\text{AGE}}_{ijk}+({a}_{11}\cdot {\text{TCLASS(0)}}_{ij}+{a}_{12}\cdot {\text{TCLASS(1)}}_{ij}+{a}_{13}\cdot {\text{TCLASS(2)}}_{ij})\cdot {\mathrm{EYEAR}}_{ij}+{b}_{i}+{\varepsilon }_{ijk}.$$

As a result of the fitting process described above, two terms from the original models (Eq. 6) were dropped: the term ${a}_{1}\cdot \mathrm{ln}({\text{AGE}}_{ijk})$ leading to a simplification of the original Hugershoff model, and the interaction term ${a}_{4}\cdot {\text{AGE}}_{ijk}\cdot {\text{EYEAR}}_{ij}$. The model contains only the random effect ${b}_{i}$ accounting for correlation at the trial level; a random effect on the plot level was not included for the same reasons as for the overarching model (Eq. 7). See Supplementary Table 10 for the parameter estimates and Fig. 3 for a visualization of model predictions.

### Supplementary Information

Supplementary Information.

## Data Availability

The data supporting the findings of this study are available from the first author upon reasonable request. See author affiliations for specific data sets.
