# Supplementary material for: Forest growth in Europe shows diverging large regional trends
Source: Sci Rep. 2023 Sep 16;13:15373. doi: 10.1038/s41598-023-41077-6 (PMC10505178; doi:10.1038/s41598-023-41077-6)
Supplement: Supplementary file 1 — Supplementary Information. [file 41598_2023_41077_MOESM1_ESM.pdf]

Supplementary Material

for

Forest Growth in Europe Shows Diverging Large Regional Trends

Hans Pretzsch, Miren del Río, Catia Arcangeli, Kamil Bielak, Malgorzata Dudzinska, David Ian Forrester, Joachim Klädtke, Ulrich Kohnle, Thomas Ledermann, Robert Matthews, Jürgen Nagel, Ralf Nagel, François Ningre, Thomas Nord-Larsen, Peter Biber

Table of Contents

Supplementary Information 1	Conversion of wood volume to biomass	2
Supplementary Table 1	Size of the data set used in this study by country, and tree species	4
Supplementary Table 2	Site characteristics of the 415 long-term experiments with 642 individual plots used in this study	5
Supplementary Table 3	Number of plots used in this study by European ecoregions and species	7
Supplementary Table 4	Condensed overview of important stand characteristics	8
Supplementary Table 5	Fit results of the species overarching non-regional growth trend model	9
Supplementary Table 6	Fit results of the non-regional growth trend model for Norway spruce	10
Supplementary Table 7	Fit results of the non-regional growth trend model for Scots pine	11
Supplementary Table 8	Fit results of the non-regional growth trend model for European beech	12
Supplementary Table 9	Fit results of the non-regional growth trend model for sessile/common oak	13
Supplementary Table 10	Fit results for the growth trends of Scots pine by trend classes	14
Supplementary Figure 1	Grid-point-wise regression slopes indicating trends in the climate-vegetation-productivity index CVP across Europe	15
Supplementary Figure 2	Overall growth trend for the main species Norway spruce, Scots pine, European beech, and sessile/common oak	16
Supplementary Figure 3	Histograms of surveys by calendar year (i.e. decade), species, and trend class	17
Supplementary Table 11	Overview of the growth and yield characteristics (last survey) of all 642 plots	18

Supplementary Information 1: Conversion of wood volume to biomass

In order to allow for comparisons of production across species, we converted the stem volume values provided by the contributing institutions into above ground woody biomass (i.e. the dry weight of the stems and branches). The backbone of our approach was to use expansion factors in the following way

$$B = EF \cdot V \cdot W \qquad \text{(Equation S1)}$$

where B and V are above ground woody biomass and stem volume. EF is a factor for expanding V to the above ground woody volume, and W is the wood density. In order to take into account tree allometry as much as possible, we used generalized volume equations provided by Forrester et al. (2017) for deriving expansion factors. These volume equations are available for estimates of the stem volume ($f(d)$) and the above ground woody volume ($g(d)$) with the stem diameter at breast height, d, as the predictor. Thus, the expansion factor for a tree with diameter d results as:

$$EF = \frac{g(d)}{f(d)} \qquad \text{(Equation S2)}$$

Both volume functions have exactly the same form:

$$f(d) = \mathrm{e}^{a_0 + a_1 \cdot d} \cdot a_2 \qquad \text{(Equation S3)}$$
$$g(d) = \mathrm{e}^{b_0 + b_1 \cdot d} \cdot b_2 \qquad \text{(Equation S4)}$$

As the species specific parameters a_0, a_1, b_0, b_1 were obtained by fitting the logarithmic forms of both equations, a_2, and b_2 are the correction factors required for making the backtransformation bias-free. The actual conversion was then straightforward, as for each plot and survey, the quadratic mean stem diameter was given as well as the volume for both, the remaining and the removed trees. This allowed us to calculate specific expansion factors after Equation S2, and subsequently apply Equation S1 to the remaining and removed stand of each plot at each survey. Our goal variable, the periodic annual biomass increment, PAI, between the survey times t_1 and t_2, resulted as

$$\mathrm{PAI} = \frac{B_{remain_2} + B_{remove_2} - B_{remain_1}}{t_2 - t_1} \qquad \text{(Equation S5)}$$

where B_{remain_1} and B_{remain_2} are the remaining biomasses at the survey times 1 and 2, and B_{remove_2} is the biomass removed at survey time 2 (or between survey times 1 and 2). From the study by Forrester et al. (2017) we took the following parameters for Equations S3 and S4; the wood densities were taken from a standard publication by Knigge and Schulz (1966).

**Species**	a_0	a_1	a_2	b_0	b_1	b_2	**wood density (kg/m^3)**
Norway spruce	-2.5027	2.3404	1.0598	-2.0464	2.3048	1.0330	377
Scots pine	-2.3583	2.3080	1.0334	-2.1212	2.2679	1.0500	431
European beech	-1.4487	2.1661	0.9979	-1.7843	2.3895	1.0920	554
sessile/common oak	-2,2131	2.3031	0.9724	-2.3149	2.5133	1.0367	561

For the species Douglas fir, and European larch, we used the parameters of Scots pine. For silver fir, we used the same parameters as for Norway spruce.

Forrester, D. I. et al. Generalized biomass and leaf area allometric equations for European tree species incorporating stand structure, tree age and climate. Forest Ecology and Management 396, 160–175 (2017).

Knigge, W.; Schulz, H. 1966: Grundriß der Forstbenutzung. Hamburg, Berlin: Parey.

Supplementary Table 1. Size of the data set used in this study by country, and tree species. The abbreviations “trls”, “plts”, “srvs” indicate the numbers of trials, plots, and surveys, respectively. Note that the overall sum of trials amounts to 420 (instead of the 415 reported in the text) because on a few trials there are plots covering different species.

**Country / Institution**	**Norway spruce**			**Scots Pine**			**Douglas fir**			**European Larch**			**silver fir**			**European beech**			**sessile/common oak**			**total**		
	**trls**	**plts**	**srvs**	**trls**	**plts**	**srvs**	**trls**	**plts**	**srvs**	**trls**	**plts**	**srvs**	**trls**	**plts**	**srvs**	**trls**	**plts**	**srvs**	**trls**	**plts**	**srvs**	**trls**	**plts**	**srvs**
Austria[1]	5	21	132	1	2	18										2	5	54				8	28	204
Denmark[2]	2	16	184										1	1	8	7	15	180	11	36	428	21	68	800
England[3]	14	27	247	21	40	352	17	17	118	9	15	121				2	2	15	13	13	102	76	114	955
France[4]																9	20	290	11	15	228	20	35	518
Germany BW[5]	57	60	490	4	4	54	3	3	20				10	10	70	19	21	154				93	98	788
Germany GOE[6]	15	25	252	9	13	120										5	11	131	14	17	131	43	66	634
Germany MUE[7]	11	18	225	14	16	149										8	12	146	8	9	76	41	55	596
Poland[8,9]				5	21	209				1	3	30				2	4	38	6	13	161	14	41	438
Switzerland[10]	11	12	104										2	2	13	6	7	90				19	21	207
Spain[11]				26	38	269																26	38	269
Sweden[12]	19	23	124	40	55	282																59	78	406
**total**	**134**	**202**	**1758**	**120**	**189**	**1453**	**20**	**20**	**138**	**10**	**18**	**151**	**13**	**13**	**91**	**60**	**97**	**1098**	**63**	**103**	**1126**	**420**	**642**	**5815**

Data provided by:

[1]Bundesforschungs- und Ausbildungszentrum für Wald, Naturgefahren und Landschaft, Wien, Austria; [2]Section Forest, Nature and Biomass, Department of Geosciences and Natural Resource Management, University of Copenhagen; [3]Forest Research, Alice Holt Lodge, Farnham, Surrey, United Kingdom; [4]Université de Lorraine, AgroParisTech, INRAE, SILVA, F-54000, Nancy, France; [5]Forstliche Versuchs- und Forschungsanstalt Baden-Württemberg, Abteilung Waldwachstum, Freiburg, Germany; [6]Nordwestdeutsche Forstliche Versuchsanstalt Sachgebiet Ertragskunde, Göttingen, Germany; [7]Chair of Forest Growth and Yield Science, School of Life Sciences Weihenstephan, Technical University of Munich, Freising, Germany; [8]Forest Research Institute, Department of Forest Management, Sekocin Stary, Poland; [9]Department of Silviculture, Institute of Forest Sciences, Warsaw University of Life Sciences, Warsaw, Poland (1 trial (5 plots) in Scots pine, 1 trial (3 plots in European Larch); [10]Swiss Federal Research Institute WSL, Birmensdorf, Switzerland; [11]INIA-CSIC Forest Research Centre, Madrid and iuFOR, Sustainable Forest Management Research Institute, University of Valladolid & INIA, Spain; [12]Swedish University of Agricultural Sciences, Alnarp and Asa, Sweden

Supplementary Table 2. Site characteristics of the 415 long-term experiments with 642 individual plots used in this study*.

**Trait**	**Class**	**Scots pine**	**Norway spruce**	**European beech**	**sessile/ common oak**	**Douglas fir**	**silver fir**	**European larch**
Annual precipitation (mm/a)	min	422	437	534	537	623	711	532
	median	598	849	746	648	1001	981	826
	max	1444	2333	1387	1160	1659	1450	939
Mean annual temperature (°C)	min	-0.3	3.3	7.6	8.0	7.4	8.1	7.9
	median	8.3	8.7	9.5	9.2	9.9	8.7	9.5
	max	12.8	10.5	11.3	11.5	11.0	10.8	10.6
Soil type<br>(Soil reference group code from the World Reference Base for Soil Resources, as provided by ESDAC)	Albeluvisol	1	1		7			
	Arenosol	18	1		1			
	Cambisol	42	119	74	55	9	10	12
	Fluvisol	2			8			4
	Gleysol	2	5		3	4	1	
	Histosol	1	2		1	1		
	Leptosol	8	6	4	3		1	
	Luvisol	13	25	14	13	3		
	Marsh		1					
	Planosol	2		5				
	Podzol	90	39		12	2		2
	Regosol	10	1				1	
	No information		2			1		
Easily available water capacity	Medium<br>(100 – 140 mm/m)	50	76	4	23	1	1	1
	High<br>(140 – 190 mm/m)	125	102	66	60	17	10	17
	Very high<br>(> 190 mm/m)	11	19	27	19		2	
	No information	3	5		1	2		
Base saturation	Low (< 50%)	135	74	8	10	4	5	2
	Medium (50 - 75%)	17	21	5	7	11	2	14
	High (> 75%)	37	105	84	86	5	6	2
	No information		2					
Soil texture	Fine<br>(35% < clay < 60%)	5	2	13	7		3	3
	Medium fine<br>(clay < 35% and sand < 15%)	11	19	27	19		2	
	Medium<br>(18% < clay < 35% and sand > 15%, or clay < 18% and 15% < sand < 65%)	120	100	53	53	17	7	14
	Coarse (clay < 18% and sand > 65%)	50	76	4	23	1	1	1
	No texture (because of organic layer)	3	2		1	1		

No information	3	1

*Annual precipitation and temperature values are averages from 1975-2014 taken from the European Commission's Agri4Cast portal (JRC 2018). All other data are soil properties from the European Soil Data Base v2 Raster Library 1 km x 1 km (Panagos et al. 2012), available from the European Soil Data Centre (ESDAC 2020). The numbers provided with the soil properties are the numbers of our plots in the respective category. In each category (soil type, easily available water capacity, base saturation, soil texture) the numbers add up to the total plot number of 642.

References for Supplementary Table 2:

JRC – Joint Research Centre of the European Commission (2018). Agri4Cast Resources Portal, available online at: https://agri4cast.jrc.ec.europa.eu/DataPortal/Index.aspx, last accessed 11 July 2018.

Panagos P., Van Liedekerke M., Jones A., Montanarella L., "European Soil Data Centre: Response to European policy support and public data requirements"; (2012) Land Use Policy, 29 (2), pp. 329-338. doi:10.1016/j.landusepol.2011.07.003

European Soil Data Centre (ESDAC), esdac.jrc.ec.europa.eu, European Commission, Joint Research Centre, last accessed 23 November 2020.

Supplementary Table 3. Number of plots used in this study by European ecoregions (alphabetical order) and species. European ecoregions after European Environment Agency (2017).

**Ecoregion**	**Scots pine**	**Norway spruce**	**European beech**	**sessile/ common oak**	**Douglas fir**	**silver fir**	**Europ. larch**	**Total**
Alps conifer and mixed forests		14						14
Baltic mixed forests		6	19	38		1		64
Caledon coniferous forests	16	4			1		2	23
Celtic broadleaf forests	18	20		7	14		6	65
Central European mixed forests	22			12			3	37
English Lowlands beech forests	6	3	2	6	2		7	26
Iberian conifer forests	11							11
Iberian sclerophyllous and semi-deciduous forests	2							2
Northen Temperate Atlantic	5	12		6				23
Northwest Iberian montane forests	25							25
Pannonian mixed forests	2		3					5
Sarmatic mixed forests	10	17						27
Scandinavian and Russian taiga	45	4						49
Southern Temperate Atlantic			7	11				18
Western European broadleaf forests	27	122	66	23	3	12		253
**Total**	189	202	97	103	20	13	18	**642**

Reference for Supplementary Table 3:

European Environment Agency (2017) Digital map of European ecological regions. Online https://www.eea.europa.eu/data-and-maps/data/digital-map-of-european-ecological-regions (last visited 8 October 2020)

Supplementary Table 4. Overview of important stand characteristics (at the last survey) of the 415 long-term experiments with 642 individual plots included in this study*.

	European beech		Douglas fir		silver fir		European larch		sessile/common oak		Scots pine		Norway spruce	
Number of plots	97		20		13		18		103		189		202	
First and last survey (year)	1870	2016	1911	2013	1883	2012	1913	2015	1885	2016	1890	2016	1873	2015
	**min**	**max**	**min**	**max**	**min**	**max**	**min**	**max**	**min**	**max**	**min**	**max**	**min**	**max**
Number of surveys	5	32	5	11	5	12	5	14	5	21	4	19	5	21
Age (a)	40	193	32	117	60	134	39	84	40	221	31	155	29	191
N (ha^{-1})	100	1600	191	1310	309	2420	388	1229	43	1143	130	2251	237	2390
dq (cm)	16,4	67,9	20,0	54,9	13,3	46,0	21,9	35,2	15,8	86,4	14,4	51,9	13,6	54,4
V (m^3 ha^{-1})	225	1209	248	1279	234	725	188	814	137	764	116	904	141	1637
PAI Volume (m^3 ha^{-1} a^{-1})	4,0	41,5	3,5	34,4	8,0	19,0	3,8	21,5	1,9	17,2	1,7	32,8	1,8	60,5
TY Volume (m^3 ha^{-1})	370	1768	415	1677	294	1154	313	949	218	1468	195	1314	234	2460
Biomass (t ha^{-1})	182	1165	121	611	124	368	91	396	133	1013	57	434	74	826
PAI Biomass (t $ha^{-1}a^{-1}$)	3,9	40,7	1,6	16,1	4,1	9,8	1,8	10,2	3,2	19,1	0,8	15,6	0,8	30,6
TY Biomass (t ha^{-1})	291	1776	204	803	156	588	152	462	209	1877	97	632	123	1246

*Abbreviations:

N: Number of trees per ha; dq: quadratic mean diameter (cm); V: standing volume (m^3 ha^{-1}); PAI Volume: periodic annual volume increment (m^3 ha^{-1} a^{-1}); TY Volume: total yield in volume (m^3 ha^{-1}), i.e. sum of standing volume and all harvested and mortality volume up to the survey of interest; Biomass: standing above ground biomass (t ha^{-1}); PAI Biomass: periodic annual increment of above ground biomass (t ha^{-1} a^{-1}); TY Biomass: total yield in biomass (t ha^{-1}), definition as for TY Volume

Supplementary Table 5. Fit results of the species overarching non-regional growth trend model (Equation 7) with 5,815 observations from 642 plots in 415 experiments*

Fixed Effect	Parameter	Estimate	Std. Error	p	sig.
	a_0	-8.8424	1.4981	0.0000	***
ln(AGE)	a_1	0.5486	0.0661	0.0000	***
AGE	a_2	0.3820	0.1615	0.0181	*
EYEAR	a_3	4.8760	0.7183	0.0000	***
AGE·EYEAR	a_4	-0.2612	0.0886	0.0032	**

Random Effects	Parameter	Std. dev.	
	b_i	τ_1	0.2571
	b_s	τ_2	0.3256
	c_s	τ_2	0.0684

	Residual	Std. dev.	
	$\varepsilon_{ijk,s}$	σ	0.3450

Significance levels '', '**', '***' correspond to $p < 0.05$, 0.01, and 0.001, respectively. Note, that in the fixed effects connected with the parameters a_2 and a_4, AGE, is the actual stand age divided by 10; in the fixed effects connected with parameters a_3 and a_4, EYEAR is the actual calendar year of stand establishment divided by 1000. These transformations improved the convergence of the model fit algorithm.

Supplementary Table 6. Fit results of the non-regional growth trend model for Norway spruce (Equation 8) with 1,758 observations from 202 plots in 134 experiments[*]

Fixed Effect	Parameter	Estimate	Std. Error	p	sig.
	a_0	-1.7709	1.2473	0.1576	
ln(AGE)	a_1	0.3194	0.0841	0.0002	***
AGE	a_2	-0.0701	0.0137	0.0000	***
EYEAR	a_3	1.6309	0.6352	0.0113	*

Random Effects	Parameter	Std. dev.	
	b_i	τ_1	0.2476
	b_{ij}	τ_4	0.0236

	Residual	Std. dev.	
	ε_{ijk}	σ	0.2876

[*]Significance levels '*', '**', '***' correspond to $p < 0.05$, 0.01, and 0.001, respectively. Note, that in the fixed effect connected with the parameter a_2, AGE, is the actual stand age divided by 10; in the fixed effect connected with parameter a_3, EYEAR is the actual calendar year of stand establishment divided by 1000. These transformations improved the convergence of the model fit algorithm.

Supplementary Table 7. Fit results of the non-regional growth trend model for Scots pine (Equation 8) with 1,453 observations from 189 plots in 120 experiments*

**Fixed Effect**	**Parameter**	**Estimate**	**Std. Error**	**p**	**sig.**
	a_0	-5.4555	1.9598	0.0061	**
ln(AGE)	a_1	0.4724	0.0890	0.0000	***
AGE	a_2	-0.1125	0.0142	0.0000	***
EYEAR	a_3	2.9702	1.0040	0.0037	**

**Random Effects**	**Parameter**	**Std. dev.**	
	b_i	τ_1	0.2714

	**Residual**	**Std. dev.**	
	ε_{ijk}	σ	0.3402

Significance levels '', '**', '***' correspond to $p < 0.05$, 0.01, and 0.001, respectively. Note, that in the fixed effect connected with the parameter a_2, AGE, is the actual stand age divided by 10; in the fixed effect connected with parameter a_3, EYEAR is the actual calendar year of stand establishment divided by 1000. These transformations improved the convergence of the model fit algorithm.

Supplementary Table 8. Fit results of the non-regional growth trend model for European beech (Equation 8) with 1,098 observations from 97 plots in 60 experiments*

**Fixed Effect**	**Parameter**	**Estimate**	**Std. Error**	**p**	**sig.**
	a_0	-10.7755	1.9281	0.0000	***
ln(AGE)	a_1	0.6905	0.1304	0.0000	***
AGE	a_2	-0.0367	0.0166	0.0275	*
EYEAR	a_3	5.5864	1.0077	0.0000	***

**Random Effects**	**Parameter**	**Std. dev.**	
	b_i	τ_1	0.2680
	b_{ij}	τ_4	0.0162

	**Residual**	**Std. dev.**	
	ε_{ijk}	σ	0.3829

Significance levels '', '**', '***' correspond to $p < 0.05$, 0.01, and 0.001, respectively. Note, that in the fixed effect connected with the parameter a_2, AGE, is the actual stand age divided by 10; in the fixed effect connected with parameter a_3, EYEAR is the actual calendar year of stand establishment divided by 1000. These transformations improved the convergence of the model fit algorithm.

Supplementary Table 9. Fit results of the non-regional growth trend model for sessile/common oak (Equation 8) with 1,126 observations from 103 plots in 63 experiments *

Fixed Effect	Parameter	Estimate	Std. Error	p	sig.
	a_0	-5.8960	1.1246	0.0000	***
ln(AGE)	a_1	0.2949	0.0341	0.0000	***
EYEAR	a_3	3.6881	0.5491	0.0000	***

Random Effects	Parameter	Std. dev.	
	b_i	τ_1	0.1355
	b_{ij}	τ_4	0.0499

	Residual	Std. dev.	
	ε_{ijk}	σ	0.3512

Significance levels '', '**', '***' correspond to $p < 0.05$, 0.01, and 0.001, respectively. Note, that in the fixed effect connected with the parameter a_2, AGE, is the actual stand age divided by 10; in the fixed effect connected with parameter a_3, EYEAR is the actual calendar year of stand establishment divided by 1000. These transformations improved the convergence of the model fit algorithm.

Supplementary Table 10. Fit results for the growth trends of Scots pine by trend classes (Equation 9)*

Fixed Effect	Parameter	Estimate	Std. Error	p	sig.
	a_0	13.5457	9.4849	0.1544	
AGE	a_2	-0.1382	0.0303	0.0000	***
EYEAR	a_3	-5.7877	4.8834	0.2370	
TCLASS(0)	a_5	-22.0814	9.7064	0.0237	*
TCLASS(1)	a_6	-16.3549	10.0050	0.1033	
TCLASS(2)	a_7	-36.9593	13.2319	0.0057	**
AGE·TCLASS(0)	a_8	0.1007	0.0310	0.0012	**
AGE·TCLASS(1)	a_9	0.0952	0.0316	0.0027	**
AGE·TCLASS(2)	a_{10}	0.1075	0.0362	0.0031	**
EYEAR·TCLASS(0)	a_{11}	11.1441	4.9995	0.0267	*
EYEAR·TCLASS(1)	a_{12}	8.2207	5.1576	0.1122	
EYEAR·TCLASS(2)	a_{13}	18.6628	6.8115	0.0067	**

Random Effects	Parameter	Std. dev.	
	b_i	τ_1	0.2108

	Residual	Std. dev.	
	ε_{ijk}	σ	0.3446

Significance levels '', '**', '***' correspond to p < 0.05, 0.01, and 0.001, respectively. Note, that AGE, is the actual stand age divided by 10, and EYEAR is the actual calendar year of stand establishment divided by 1000. These transformations improved the convergence of the model fit algorithm.

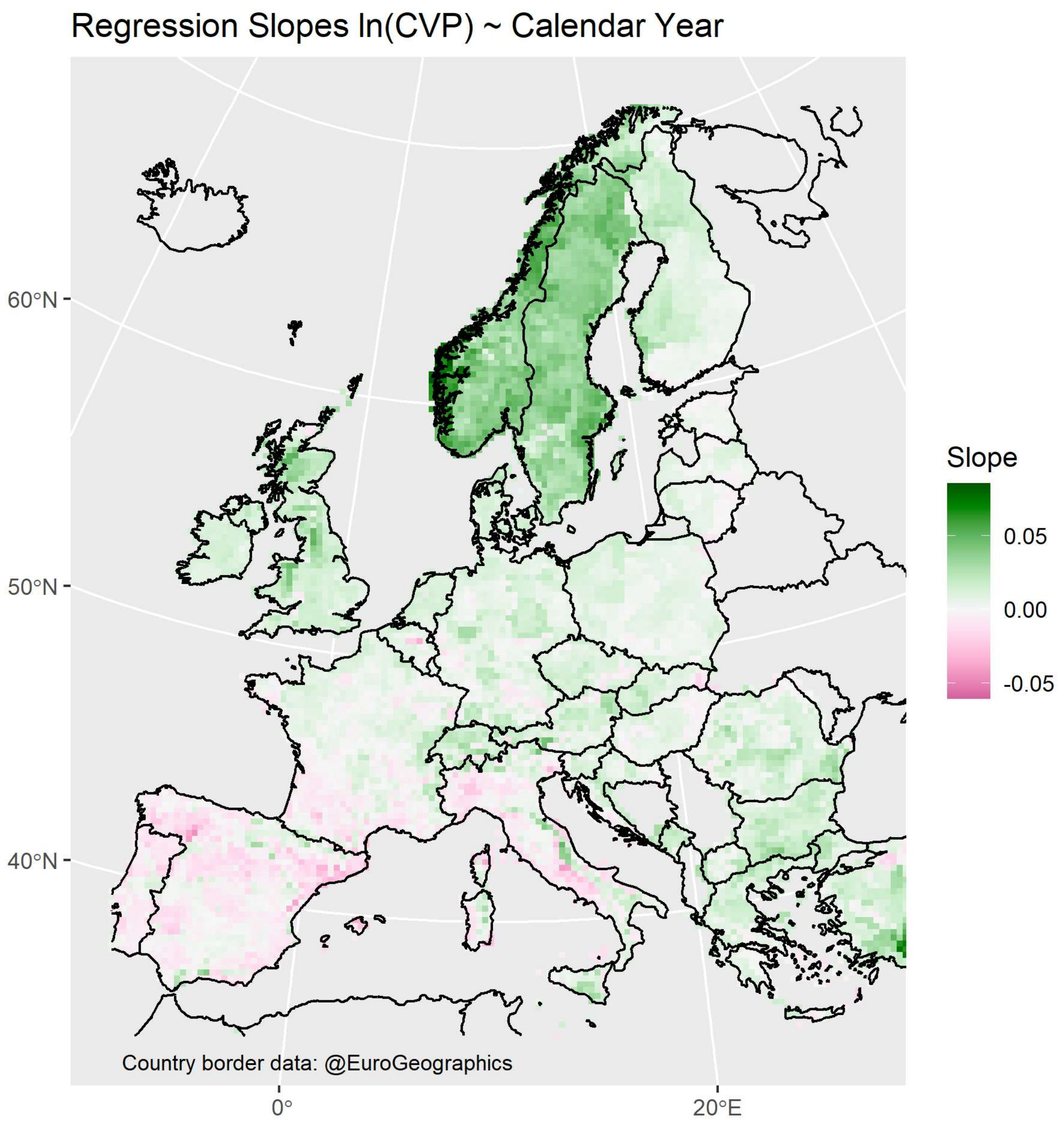

Supplementary Figure 1. Grid-point-wise regression slopes (parameter a_1 in Equation 3) indicating trends in the climate-vegetation-productivity index CVP across Europe in the period of 1975-2017 on a 25 x 25 km raster. Positive slopes indicate a trend towards more favourable climate conditions for forest growth; negative slopes indicate deteriorating conditions. These slope values were the basis for defining the trend classes shown in Figure 2.

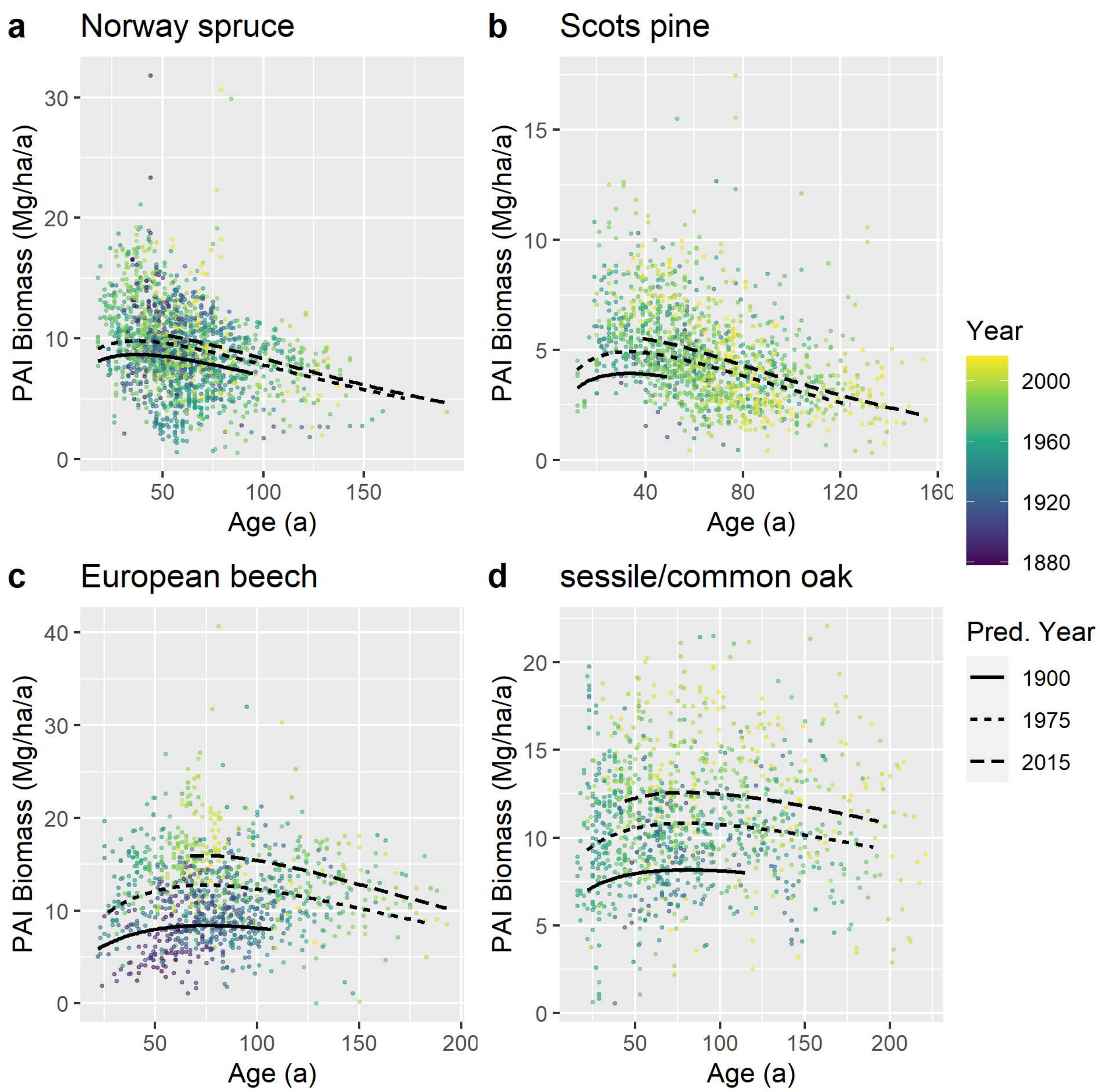

Supplementary Figure 2. Overall growth trend for the main species Norway spruce (a, 1758 observations, 202 plots, 134 trials), Scots pine (b, 1453 obs., 189 plots, 120 trials), European beech (c, 1098 obs., 97 plots, 60 trials) and sessile/common oak (d, 1126 obs., 103 plots, 63 trials). The model lines are estimates based on the fitted fixed effects of Equation 8 (see Supplementary Tables 6-9). Note the unimodal shape of the estimates for oak, even if there is only a positive age effect in the fitted model (Supplementary Table 9). This occurs because each model line relates to stands at different ages in one specific prediction year, not to one stand getting older.

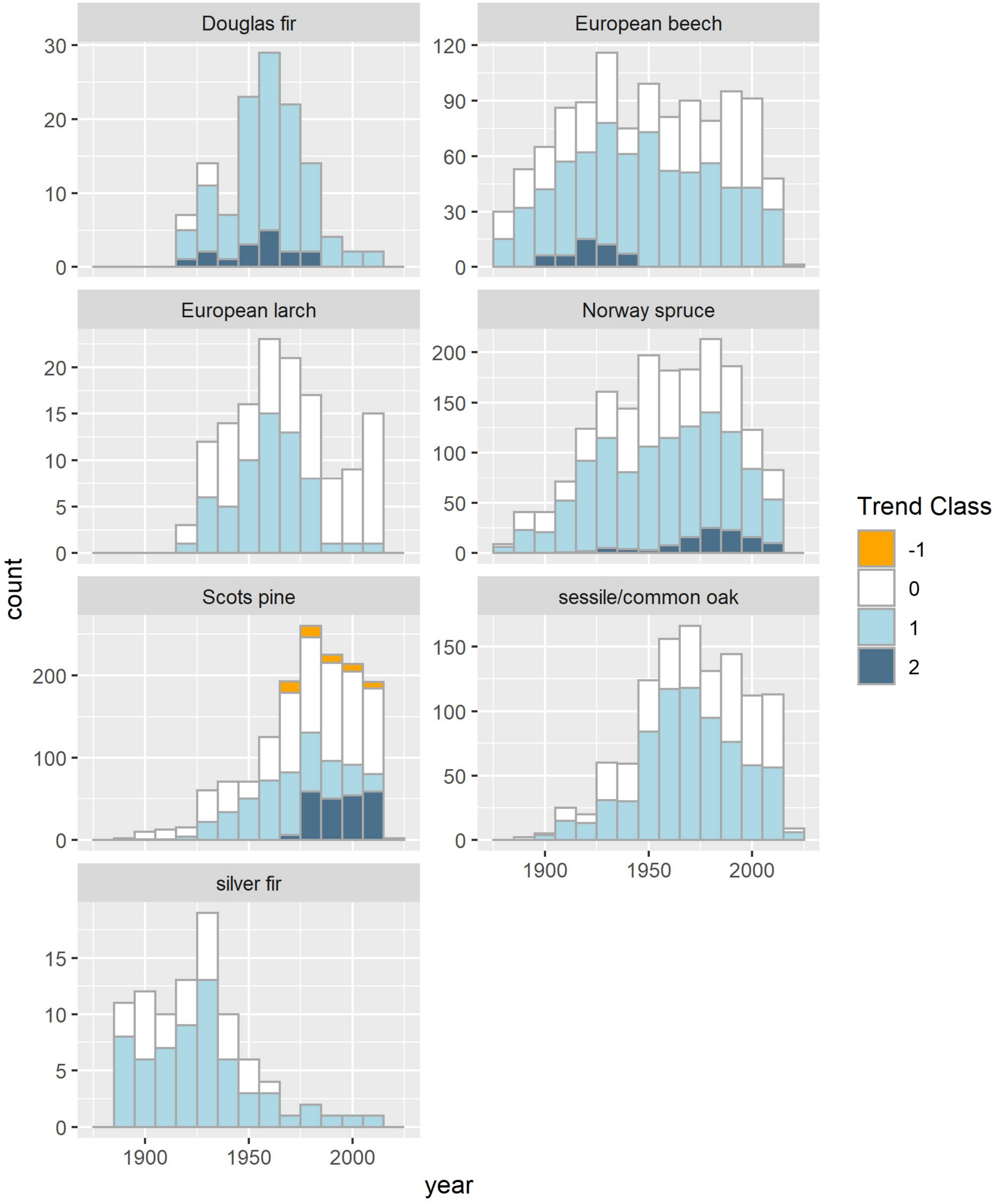

Supplementary Figure 3. Histograms of surveys by calendar year (i.e. decade), species, and trend class. The trend classes are colour-coded as in Figure 2. Numbers of surveys: Douglas fir, 138; European beech, 1098; European larch, 151; Norway spruce, 1758; Scots pine, 1453; sessile/common oak, 1126; silver fir, 91.

Supplementary Table 11. Overview of the growth and yield characteristics (last survey) of all 642 plots, sorted by species. See Supplementary Table 1 for the names of the data-providing instutions per country. Abbreviations: N: Number of trees per ha; dq: quadratic mean diameter (cm); V: standing volume (m³ ha^{-1}); PAI Volume: periodic annual volume increment (m³ ha^{-1} a^{-1}); TY Volume: total yield in volume (m³/ha), i.e. sum of standing volume and all harvested and mortality volume up to the survey of interest; Biomass: standing above ground biomass (t/ha); PAI Biomass: periodic annual increment of above ground biomass (t ha^{-1} a^{-1}); TY Biomass: total yield in biomass (t ha^{-1}), definition as for TY Volume

Species	Country / Institution	Trial/Plot	Age at last survey (a)	First survey (year)	Last survey (year)	Number of surveys	N (ha^{-1})	dq (cm)	SDI	V (m^3 ha^{-1})	PAI Volume (m^3 ha^{-1} a^{-1})	TY Volume (m^3 ha^{-1})	Biomass (t ha^{-1})	PAI Biomass i (t ha^{-1} a^{-1})	TY Biomass (t ha^{-1})
European beech	Austria	204/1	103	1895	1943	17	460	30.5	633	394	6.0	666	367	7.6	602
European beech	Austria	204/2	99	1895	1939	16	468	31.4	675	421	9.0	697	394	9.6	633
European beech	Austria	204/3	99	1895	1939	16	332	33.9	541	353	9.0	696	336	10.2	637
European beech	Austria	315/13	66	1996	2014	5	1308	20.8	974	540	14.2	605	461	14.4	515
European beech	Austria	315/33	66	1996	2014	5	1600	18.4	978	480	16.0	531	399	15.5	440
European beech	Denmark	D$/1	71	1956	1978	6	169	43.0	404	430	20.3	749	432	22.2	739
European beech	Denmark	DA/1	134	1903	1977	20	370	39.7	779	936	15.5	1158	924	16.8	1136
European beech	Denmark	DEX/1	188	1946	1981	9	135	60.2	552	820	9.4	1186	888	12.2	1281
European beech	Denmark	DP/10	97	1938	2015	32	949	28.3	1158	1013	16.2	1221	927	17.3	1105
European beech	Denmark	DP/11	80	1938	1998	18	529	30.5	728	668	5.9	1063	622	6.1	957
European beech	Denmark	DP/3	63	1938	1981	13	644	25.4	658	470	19.0	787	419	18.5	678
European beech	Denmark	DP/5	49	1938	1967	10	886	19.3	586	310	21.9	509	261	20.5	416
European beech	Denmark	DQ/A1	46	1941	1962	10	798	19.0	512	267	18.7	463	223	16.9	377
European beech	Denmark	DQ/A2	43	1941	1959	9	858	17.7	494	239	16.9	387	197	15.2	312
European beech	Denmark	DQ/D1	43	1941	1959	9	747	19.0	480	240	16.8	411	201	15.5	335
European beech	Denmark	DQ/D2	40	1941	1956	8	997	16.4	508	225	17.6	370	182	14.8	291
European beech	Denmark	DQ/E2	84	1941	2000	20	821	28.7	1024	900	7.8	1051	826	9.0	954
European beech	Denmark	DQ/E3	81	1941	1997	12	986	30.8	1380	1209	41.5	1281	1127	40.7	1191
European beech	Denmark	DV/2	119	1938	1989	12	440	34.9	750	813	25.5	1446	780	25.3	1378
European beech	Denmark	S/1	118	1887	1930	8	162	47.2	449	542	13.1	926	556	14.6	931

European beech	England	Nettlebed - Lower Common Wood/1256	138	1949	1991	8	290	38.9	588	409	7.0	506	402	7.2	494
European beech	England	Queen & College Wood/1373	193	1953	2006	9	129	51.0	405	345	7.5	591	360	8.5	603
European beech	France	CampCusson/2	101	1924	1981	12	248	41.6	561	572	8.7	926	571	10.9	900
European beech	France	CampCusson/4	97	1924	1977	10	275	42.2	637	653	11.3	1078	653	12.2	1047
European beech	France	Charlemagne/1	134	1904	1989	17	232	50.1	707	934	14.6	1492	971	16.7	1513
European beech	France	Charlemagne/3	151	1904	2006	20	304	46.5	822	1076	13.0	1768	1100	14.4	1776
European beech	France	Chavigny/1	138	1904	1995	18	200	51.8	643	857	11.2	1466	898	13.0	1495
European beech	France	Chavigny/2	109	1904	1966	13	240	44.6	608	685	12.8	1080	694	14.4	1065
European beech	France	Chretiennette/2	118	1922	1978	10	188	51.4	598	797	10.1	1183	834	11.7	1212
European beech	France	Chretiennette/3	118	1922	1978	10	252	46.1	673	830	9.8	1223	847	11.4	1241
European beech	France	Epiceas/1	150	1904	2006	22	184	55.3	658	954	16.0	1541	1014	18.1	1587
European beech	France	Epiceas/3	150	1904	2006	22	328	46.6	891	1139	15.1	1597	1165	16.9	1603
European beech	France	Faite/2	91	1922	1978	12	440	31.2	627	518	11.4	810	484	11.6	734
European beech	France	Faite/3	68	1922	1955	9	970	20.7	715	389	18.3	533	332	16.7	448
European beech	France	PreDesSeigneurs/1	173	1922	1968	9	100	67.9	497	846	11.4	1423	942	13.8	1561
European beech	France	SouillyB/1	81	1963	2009	18	580	32.1	868	736	9.9	890	693	10.6	830
European beech	France	SouillyB/3	81	1963	2009	18	515	34.0	845	753	14.0	908	718	15.4	858
European beech	France	SouillyB/4	81	1963	2009	18	710	30.2	961	789	15.4	913	732	16.6	841
European beech	France	SouillyD/1	81	1963	2009	18	520	31.0	733	615	20.7	849	575	20.6	777
European beech	France	SouillyD/2	81	1963	2009	18	470	32.5	715	616	18.4	857	581	18.7	794
European beech	France	SouillyD/3	81	1963	2009	18	475	32.2	712	610	16.6	867	574	17.3	800
European beech	France	SouillyD/4	81	1963	2009	18	470	32.8	728	634	19.2	953	600	19.8	883
European beech	Germany BW	Bu 9/1	97	1874	1925	7	584	22.9	507	262	6.8	426	229	6.4	365
European beech	Germany BW	Bu 13/1	98	1875	1926	9	672	21.7	535	254	9.1	391	219	8.7	326
European beech	Germany BW	Bu 17/1	109	1875	1922	7	392	30.5	539	462	10.8	752	430	10.9	687
European beech	Germany BW	Bu 18/1	109	1875	1922	7	372	31.3	534	458	12.0	736	429	12.1	678
European beech	Germany BW	Bu 21/1	97	1875	1930	9	648	22.4	543	274	7.3	466	238	7.1	390
European beech	Germany BW	Bu 22/1	93	1875	1926	8	628	23.7	576	315	8.6	485	277	8.3	418
European beech	Germany BW	Bu 25/1	112	1875	1953	13	370	31.2	528	378	6.7	623	353	6.7	564

European beech	Germany BW	Bu 31/1	104	1875	1928	9	312	38.3	619	584	17.8	970	572	19.3	922
European beech	Germany BW	Bu 35/1	105	1875	1927	8	380	31.6	553	412	10.3	642	386	10.7	590
European beech	Germany BW	Bu 66/1	92	1876	1922	7	704	22.6	599	325	10.1	538	283	9.3	462
European beech	Germany BW	Bu 91/1	105	1876	1932	9	428	31.2	611	453	9.5	734	424	10.3	672
European beech	Germany BW	Bu 101/1	71	1876	1906	5	476	28.4	584	400	9.7	555	366	10.0	503
European beech	Germany BW	Bu 107/1	111	1877	1935	12	412	34.0	675	621	12.4	1003	592	13.3	929
European beech	Germany BW	Bu 113/1	113	1877	1930	9	544	28.2	660	487	9.5	788	445	10.1	700
European beech	Germany BW	Bu 123/1	121	1877	1930	9	288	36.5	529	451	9.0	782	437	10.0	737
European beech	Germany BW	Bu 132/1	102	1877	1928	8	376	28.9	474	329	11.3	568	302	11.3	511
European beech	Germany BW	Bu 153/1	96	1877	1913	7	464	27.4	538	339	9.5	563	308	9.2	502
European beech	Germany BW	Bu 162/1	101	1877	1922	7	624	23.8	577	307	6.5	462	270	6.1	397
European beech	Germany BW	Bu 253/1	93	1889	1940	9	608	23.1	536	323	9.7	493	283	9.1	421
European beech	Germany BW	Bu 253/2	93	1884	1940	10	428	27.4	496	342	8.8	566	310	8.8	504
European beech	Germany BW	Bu 253/3	78	1884	1925	6	488	23.6	445	245	11.3	385	215	11.0	333
European beech	Germany GOE	ID 042210/2	140	1890	1982	16	300	41.2	669	709	13.2	958	706	13.9	930
European beech	Germany GOE	ID 042210/5	130	1930	1972	9	348	36.2	630	593	10.5	731	573	11.3	703
European beech	Germany GOE	ID 042210/6	145	1950	1987	8	144	54.9	509	647	5.6	960	687	7.6	1005
European beech	Germany GOE	ID 042210/7	145	1950	1987	8	168	50.6	521	636	10.4	974	663	12.5	1000
European beech	Germany GOE	ID 080210/2	109	1951	2004	12	400	33.1	628	464	7.8	744	440	8.2	689
European beech	Germany GOE	ID 080210/3	117	1951	2012	14	687	29.7	906	701	13.2	938	648	13.0	857
European beech	Germany GOE	ID 583210/3	122	1898	1968	13	610	33.0	952	871	20.3	1147	825	20.7	1074
European beech	Germany GOE	ID H10210/1	168	1886	2014	23	217	48.9	637	907	12.8	1398	938	14.1	1413
European beech	Germany GOE	ID H10210/2	147	1886	1993	18	184	47.8	521	657	7.2	1156	676	8.3	1143
European beech	Germany GOE	ID J51210/1	68	1976	2014	11	1474	19.8	1014	534	13.2	619	451	12.6	521
European beech	Germany GOE	ID J51210/5	68	1976	2014	11	1578	19.4	1050	529	11.2	580	445	10.6	487
European beech	Germany MUE	111/4	132	1954	2008	9	296	37.4	565	627	13.8	875	611	14.4	838
European beech	Germany MUE	43845	188	1870	2010	18	378	44.1	940	1107	8.2	1434	1119	9.1	1422
European beech	Germany MUE	43876	178	1870	2000	17	219	51.5	699	924	10.1	1438	967	12.4	1456
European beech	Germany MUE	232/4	128	1961	2015	10	187	49.0	551	635	5.5	1066	657	6.6	1071

European beech	Germany MUE	43855	153	1870	1981	15	356	36.9	665	593	10.2	884	576	11.2	830
European beech	Germany MUE	43886	164	1870	1992	16	292	38.4	581	507	9.6	846	497	10.2	808
European beech	Germany MUE	43856	144	1871	1967	14	425	37.0	797	796	10.8	1046	773	11.4	998
European beech	Germany MUE	43887	144	1871	1967	14	303	39.9	642	678	11.2	1069	670	11.9	1027
European beech	Germany MUE	43857	182	1881	2014	18	272	47.3	757	962	8.1	1348	987	9.0	1347
European beech	Germany MUE	43888	152	1881	1984	15	219	45.3	569	706	9.3	1203	718	10.2	1179
European beech	Germany MUE	312/2	89	1959	1984	5	596	23.9	554	316	13.3	493	278	12.7	428
European beech	Germany MUE	91/4	85	1971	2012	7	709	25.8	748	569	18.3	865	510	17.3	761
European beech	Poland	SCH_E/33	108	1890	1933	7	204	41.1	453	423	9.5	725	421	10.5	714
European beech	Poland	SCH_E/34	116	1890	1941	9	212	39.6	444	402	13.3	732	397	14.4	709
European beech	Poland	SCH_F/38	120	1929	2016	16	257	45.1	661	659	9.6	980	669	10.3	953
European beech	Poland	SCH_F/39	90	1929	1986	10	400	32.3	603	439	12.2	665	414	12.8	609
European beech	Switzerland	2019000/1	120	1907	2009	17	445	39.2	915	743	11.1	1051	732	11.9	1010
European beech	Switzerland	41014000/1	124	1890	1991	16	296	43.3	716	656	14.3	972	661	16.0	942
European beech	Switzerland	41018000/1	93	1890	1954	12	528	24.5	510	262	4.0	432	232	3.9	376
European beech	Switzerland	4102/2	91	1890	1946	12	560	26.9	629	345	6.0	580	312	6.0	513
European beech	Switzerland	4102/4	133	1890	1986	16	320	43.5	777	625	11.9	911	630	13.1	895
European beech	Switzerland	41036000/1	87	1892	1950	11	624	25.5	645	310	6.2	414	277	6.2	363
European beech	Switzerland	41193000/1	142	1905	1991	13	275	43.6	672	586	12.4	889	591	13.8	877
Douglas fir	England	Achnashellach/3256	57	1951	1982	9	318	45.5	833	580	20.8	969	276	9.7	464
Douglas fir	England	Bodmin - Trengoffe/1191	65	1947	1992	11	210	53.0	702	697	21.4	1029	330	10.0	491
Douglas fir	England	Cardinham/1195	71	1947	1993	11	191	51.8	615	577	34.4	893	273	16.1	426
Douglas fir	England	Cardinham_2/1199	44	1947	1968	7	517	30.5	710	351	17.3	593	170	8.2	289
Douglas fir	England	Cruach Wood/3098	76	1932	1954	7	247	47.4	690	647	33.0	1097	307	15.6	522
Douglas fir	England	Culloden/3080	46	1926	1951	6	1134	24.9	1129	544	19.3	722	265	9.2	353
Douglas fir	England	Diosgydd/2035	62	1948	1982	9	214	54.9	757	720	27.9	1108	340	13.0	527
Douglas fir	England	Dodd Wood/1264	32	1950	1962	5	725	23.4	654	248	20.8	415	121	10.0	204
Douglas fir	England	Dunster - Aville Wood/1044	61	1921	1955	8	463	41.4	1040	804	30.6	1027	384	14.5	491
Douglas fir	England	Dunster - Broad Wood/1018	67	1912	1946	8	291	44.6	736	627	19.3	1061	298	9.0	507

Douglas fir	England	Lake Vyrnwy/2010	44	1920	1940	5	610	29.6	799	447	14.7	735	217	7.0	357
Douglas fir	England	Monaughty/3183	47	1948	1974	9	349	40.8	767	571	27.5	992	273	13.0	477
Douglas fir	England	Mynydd Du/2163	32	1959	1976	5	417	32.0	619	349	29.7	565	168	14.1	274
Douglas fir	England	Side Wood/1218	44	1947	1972	7	330	35.7	584	406	26.1	613	195	12.4	296
Douglas fir	England	Snoring/1297	49	1952	1983	7	1267	25.2	1287	630	25.2	718	307	12.1	350
Douglas fir	England	Tortworth - Ironmill Grove/1019	117	1918	1985	11	333	49.1	985	975	3.5	1260	463	1.6	598
Douglas fir	England	Tortworth - Ironmill Grove_2/1020	103	1918	1985	11	292	45.8	772	649	6.2	748	309	2.9	356
Douglas fir	Germany BW	Dgl 5/1	41	1911	1931	6	960	22.6	816	450	19.7	805	220	9.4	395
Douglas fir	Germany BW	Dgl 7/1	39	1911	1931	6	1310	20.0	916	442	24.0	731	217	11.5	361
Douglas fir	Germany BW	Dgl 98/1	81	1957	2013	11	559	41.9	1280	1279	29.1	1677	611	13.7	803
Silver fir	Denmark	OD/1	134	1954	2012	9	550	38.3	1091	514	14.0	611	262	7.1	312
Silver fir	Germany BW	Ta 71/1	91	1883	1922	6	1125	21.2	863	427	14.3	724	222	7.3	378
Silver fir	Germany BW	Ta 72/1	101	1883	1933	8	860	24.2	816	459	16.9	803	238	8.6	418
Silver fir	Germany BW	Ta 73/1	104	1883	1933	8	785	25.2	795	465	15.9	817	241	8.1	425
Silver fir	Germany BW	Ta 74/1	108	1883	1938	9	727	27.5	847	519	13.0	916	268	6.5	475
Silver fir	Germany BW	Ta 89/1	75	1897	1943	10	640	29.5	835	597	15.0	1015	307	7.6	525
Silver fir	Germany BW	Ta 219/B	68	1924	1949	5	1380	20.2	980	446	19.0	576	232	9.8	301
Silver fir	Germany BW	Ta 229/1	60	1883	1904	5	2420	13.3	879	234	8.0	294	124	4.1	156
Silver fir	Germany BW	Ta 233/1	76	1890	1941	8	872	25.7	912	469	15.5	768	242	7.8	398
Silver fir	Germany BW	Ta 239/1	96	1887	1944	9	393	37.5	753	598	9.7	1042	305	4.8	535
Silver fir	Germany BW	Ta 242/1	85	1898	1960	12	440	33.4	700	502	10.9	779	257	5.4	401
Silver fir	Switzerland	1014000/1	133	1905	1944	8	309	46.0	823	671	11.6	1141	339	5.7	579
Silver fir	Switzerland	1030003/1	114	1914	1964	7	342	43.5	831	725	11.9	1154	368	5.9	588
European larch	England	Bennachie - Kemnay/3180	80	1948	2009	13	704	29.5	917	583	9.7	772	282	4.6	375
European larch	England	Bolitho's Plantation/1034	75	1920	1967	11	618	27.3	711	511	18.1	778	248	8.7	380
European larch	England	Kidnalls/1415	44	1956	1977	6	503	27.4	583	295	14.8	486	143	7.0	237
European larch	England	Kidnalls/1416	78	1956	2012	12	1229	26.0	1311	814	8.6	948	396	4.1	462
European larch	England	Murthly - Duncans Hill/3045	70	1921	1967	11	862	25.3	876	475	6.6	582	232	3.2	284

European larch	England	Reddings Lodge/1105	65	1929	1974	10	649	29.4	842	515	5.2	643	249	2.4	312
European larch	England	Reddings Lodge/1108	65	1929	1974	10	571	30.5	784	526	12.2	663	254	5.8	321
European larch	England	Reddings Lodge/1125	40	1929	1949	5	476	26.3	518	233	13.5	366	113	6.5	179
European larch	England	Reddings Lodge/1127	39	1929	1949	5	476	26.5	522	226	11.2	344	110	5.3	168
European larch	England	Seafield - Tom-An-Uird/3067	77	1924	1970	10	826	21.9	667	314	3.8	414	154	1.8	203
European larch	England	Shambellie Wood/3074	84	1926	1994	14	481	35.2	833	610	6.1	949	293	2.8	459
European larch	England	The Oaks/2003	61	1913	1938	6	582	24.3	558	275	7.4	407	134	3.5	199
European larch	England	Wigmore Rolls/1400	47	1954	1982	8	388	29.5	505	188	6.3	313	91	2.9	152
European larch	England	Wigmore Rolls/1401	47	1954	1982	8	482	26.3	522	188	4.9	316	91	2.2	154
European larch	England	Wigmore Rolls/1402	47	1954	1982	8	586	24.6	570	217	8.3	344	106	3.9	169
European larch	Poland	Rogow/1	51	1975	2015	11	463	34.1	761	575	21.5	770	277	10.2	372
European larch	Poland	Rogow/2	51	1975	2015	11	460	33.2	724	534	14.7	717	258	7.0	347
European larch	Poland	Rogow/3	51	1975	2015	11	514	32.2	770	559	16.4	745	270	7.8	361
sessile / common Oak	Denmark	AZ/1	112	1893	1930	6	190	41.4	427	387	5.4	586	457	7.3	684
sessile / common Oak	Denmark	BN/1	96	1894	1930	6	194	38.0	381	321	7.6	522	373	9.5	596
sessile / common Oak	Denmark	CS/1	184	1903	1969	16	43	86.4	314	548	9.3	853	755	13.4	1162
sessile / common Oak	Denmark	CT/1	98	1903	1930	6	69	52.7	228	247	7.9	383	307	10.6	471
sessile / common Oak	Denmark	QD/3	121	1920	2014	19	267	41.4	599	598	9.7	938	707	14.2	1071
sessile / common Oak	Denmark	QR/1	201	1936	2015	11	79	71.2	426	587	6.3	819	777	8.9	1061
sessile / common Oak	Denmark	QS/1	180	1931	2011	11	46	85.4	329	577	2.5	803	794	3.7	1090
sessile / common Oak	Denmark	QX/F1	85	1945	2009	17	571	30.2	774	596	10.8	666	659	13.0	733
sessile / common Oak	Denmark	QX/F2	85	1945	2009	16	497	29.9	664	528	10.8	651	583	13.4	716
sessile / common Oak	Denmark	QX/G1	85	1945	2009	17	355	32.4	538	450	10.7	739	504	13.0	803
sessile / common Oak	Denmark	QX/G2	85	1945	2009	17	379	31.7	554	449	10.5	725	502	12.7	785
sessile / common Oak	Denmark	QX/K1	85	1945	2009	17	322	34.8	546	434	9.4	718	494	11.8	790
sessile / common Oak	Denmark	QX/K2	85	1945	2009	17	296	36.4	541	456	10.0	761	524	12.6	843
sessile / common Oak	Denmark	QX/L1	85	1945	2009	16	513	31.3	735	570	9.5	677	635	11.8	750
sessile / common Oak	Denmark	QX/L2	85	1945	2009	17	609	30.9	854	664	12.7	752	737	15.5	832
sessile / common Oak	Denmark	QY/N1	85	1947	2009	15	363	32.5	553	429	7.5	696	482	9.2	759

sessile / common Oak	Denmark	QY/N2	71	1947	1995	14	336	32.2	503	394	13.0	639	441	15.8	695
sessile / common Oak	Denmark	QY/Q1	85	1947	2009	15	292	35.8	521	456	8.7	739	523	11.0	821
sessile / common Oak	Denmark	QY/Q2	85	1947	2009	15	302	34.9	514	450	8.2	749	513	10.3	828
sessile / common Oak	Denmark	QY/T1	85	1947	2009	15	473	31.3	677	536	9.9	731	597	12.3	808
sessile / common Oak	Denmark	QY/T2	90	1947	2014	16	312	36.9	583	505	10.1	773	582	14.9	883
sessile / common Oak	Denmark	QZ/1	119	1945	2012	11	62	63.4	276	336	2.6	547	435	4.1	685
sessile / common Oak	Denmark	QZ/2	85	1945	1978	8	88	46.9	243	242	6.7	391	293	8.8	466
sessile / common Oak	Denmark	QZ/3	78	1945	1971	8	90	46.5	243	238	7.9	364	288	10.5	434
sessile / common Oak	Denmark	QZ/4	119	1945	2012	12	57	65.9	269	343	3.9	554	446	5.7	698
sessile / common Oak	Denmark	QZ/5	121	1945	2012	12	63	64.3	289	366	4.8	568	475	6.9	715
sessile / common Oak	Denmark	QZ/6	87	1945	1978	9	79	50.0	241	251	6.1	410	309	8.1	494
sessile / common Oak	Denmark	QZ/7	94	1945	1985	10	63	55.6	227	259	6.5	437	325	9.0	537
sessile / common Oak	Denmark	QZ/8	94	1945	1985	10	62	57.4	236	278	7.4	476	352	10.2	588
sessile / common Oak	Denmark	QZ/9	94	1945	1985	10	67	57.8	256	309	6.9	509	392	9.7	630
sessile / common Oak	Denmark	RA/1	72	1958	2016	16	496	25.8	522	356	5.0	592	381	8.6	612
sessile / common Oak	Denmark	RA/10	72	1958	2016	16	626	25.6	648	427	3.7	608	456	4.7	635
sessile / common Oak	Denmark	RA/11	52	1958	1996	11	645	22.4	539	334	17.2	532	347	19.1	524
sessile / common Oak	Denmark	RA/12	72	1958	2016	16	588	26.2	635	459	8.3	606	492	10.4	638
sessile / common Oak	Denmark	RA/2	52	1958	1996	11	612	23.1	540	318	16.1	535	332	18.2	534
sessile / common Oak	Denmark	RA/5	58	1958	2002	12	515	24.4	495	319	8.5	543	337	10.2	547
sessile / common Oak	England	Abbotswood/1413	51	1956	1978	5	705	19.7	480	195	13.7	326	197	15.1	322
sessile / common Oak	England	Apethorpe/1249	55	1949	1974	7	535	21.5	421	153	7.3	244	158	8.1	246
sessile / common Oak	England	Batty Daws Wood/1029	153	1920	2010	14	51	66.8	247	252	6.4	696	330	8.9	861
sessile / common Oak	England	Didlington/1307	82	1952	2010	12	462	30.6	638	378	10.6	536	419	12.3	582
sessile / common Oak	England	Dymock Wood/1030	113	1920	1974	10	245	38.3	486	298	12.9	474	346	15.7	540
sessile / common Oak	England	Fineshade. Town Wood/1344	57	1953	1985	7	461	24.8	455	191	7.3	312	202	8.3	324
sessile / common Oak	England	Hazelborough Green Man/1364	84	1953	2013	13	458	29.4	595	357	9.2	552	393	12.2	599
sessile / common Oak	England	High Meadow - Knockalls/1095	40	1927	1950	5	926	16.2	459	137	8.8	218	133	9.2	209
sessile / common Oak	England	Micheldever/1320	86	1952	2013	14	479	31.0	677	401	2.4	573	446	3.6	627

sessile / common Oak	England	Roudham - High Bridgham/1436	49	1956	1982	7	483	22.8	415	156	8.5	266	163	9.7	270
sessile / common Oak	England	Snoring/1300	53	1952	1988	8	624	23.2	553	231	6.9	326	242	7.7	333
sessile / common Oak	England	Wensum - Swanton Novers/1303	48	1952	1978	6	577	20.7	426	166	8.4	289	169	9.5	289
sessile / common Oak	England	Wyre Forest/1271	104	1950	1991	7	159	47.4	444	323	6.7	458	393	8.7	545
sessile / common Oak	France	Belleme chatelier/1	177	1934	2016	15	130	61.6	553	760	8.9	1128	976	12.4	1420
sessile / common Oak	France	Blois Allee-de-Blois/Allee-de-Blois	208	1927	2014	17	131	60.3	538	751	10.5	1165	960	14.3	1455
sessile / common Oak	France	Blois Marchais-Des-Cordeliers/1	189	1925	2014	19	158	54.6	553	720	12.2	1157	902	16.5	1410
sessile / common Oak	France	Blois Pauvert/1	153	1926	2014	18	204	46.2	547	617	10.5	1090	746	13.9	1269
sessile / common Oak	France	Blois Sablonnieres/2B	125	1925	2014	16	377	35.6	664	652	14.5	959	746	17.9	1067
sessile / common Oak	France	Blois Sablonnieres/3	125	1925	2014	15	278	39.2	573	569	10.7	981	665	13.6	1086
sessile / common Oak	France	Champenoux Butte-de-Tir/2	130	1928	2015	19	284	38.8	574	545	11.3	1039	635	14.1	1150
sessile / common Oak	France	Champenoux Grande Bouzule/2	147	1928	2015	21	206	46.5	557	611	9.2	1014	739	12.2	1199
sessile / common Oak	France	Reno-Valdieu Reno/C12	99	1956	2015	16	305	38.7	614	613	12.9	844	714	16.1	953
sessile / common Oak	France	Reno-Valdieu Reno/C14	99	1956	2015	16	330	36.1	595	577	14.1	784	662	17.4	871
sessile / common Oak	France	Reno-Valdieu Reno/C3	99	1956	2015	16	420	32.5	641	550	15.2	924	617	18.3	990
sessile / common Oak	France	Reno-Valdieu Reno/C5	99	1956	2015	15	435	31.1	619	486	13.0	799	541	15.4	846
sessile / common Oak	France	Troncais Cles-des-Fosses/Cles-des-Fosses	195	1931	2016	15	88	72.8	489	761	9.1	1468	1013	13.1	1877
sessile / common Oak	France	Troncais Richebourg/Richebourg	202	1931	2003	11	81	73.7	457	703	7.7	1119	938	11.4	1469
sessile / common Oak	France	Troncais Tresor/1	137	1932	2016	15	263	43.3	634	732	12.6	1147	873	16.2	1322
sessile / common Oak	Germany GOE	ID 012103/2	168	1931	2005	15	192	62.8	842	686	12.8	1108	885	17.6	1413
sessile / common Oak	Germany GOE	ID 033122/2	162	1930	1992	12	160	48.9	470	541	7.3	915	662	10.1	1099
sessile / common Oak	Germany GOE	ID 052122/2	214	1929	2009	16	282	53.4	953	492	8.6	833	613	11.2	1029
sessile / common Oak	Germany GOE	ID 640110/1	60	1983	2010	7	529	26.7	588	360	10.6	473	388	12.9	504
sessile / common Oak	Germany GOE	ID 642120/1	61	1983	2010	7	788	24.9	783	497	16.2	614	528	18.7	645
sessile / common Oak	Germany GOE	ID 643110/2	63	1983	2010	7	558	25.8	587	341	10.8	427	365	12.9	454
sessile / common Oak	Germany GOE	ID 644110/3	63	1983	2011	7	540	26.0	575	358	9.5	481	384	11.2	509
sessile / common Oak	Germany GOE	ID 645110/1	65	1983	2010	7	562	24.4	541	299	12.4	416	316	13.9	432

sessile / common Oak	Germany GOE	ID 646110/1	66	1983	2010	7	521	28.5	643	415	11.8	494	453	14.2	537
sessile / common Oak	Germany GOE	ID 778110/3	185	1929	2013	15	102	67.6	503	660	9.4	969	865	13.1	1257
sessile / common Oak	Germany GOE	ID 858120/1	44	1988	2012	6	1143	15.8	547	184	7.0	236	178	8.1	227
sessile / common Oak	Germany GOE	ID 858120/10	44	1988	2012	6	776	19.5	521	219	8.4	305	221	10.6	306
sessile / common Oak	Germany GOE	ID 905110/6	43	1991	2011	6	894	19.4	595	258	12.0	333	260	13.9	333
sessile / common Oak	Germany GOE	ID H34120/2	61	1976	2010	9	1087	20.0	760	386	12.0	458	391	13.5	462
sessile / common Oak	Germany GOE	ID H34120/4	61	1976	2010	9	1057	21.4	824	458	15.8	573	471	18.1	584
sessile / common Oak	Germany GOE	ID H36110/3	42	1989	2013	6	922	20.3	660	345	15.2	443	351	16.8	447
sessile / common Oak	Germany GOE	ID H36110/5	42	1989	2013	6	875	20.5	636	336	14.7	426	343	16.2	431
sessile / common Oak	Germany MUE	311/1	113	1959	2014	9	342	37.3	650	535	12.5	827	619	15.5	928
sessile / common Oak	Germany MUE	39/1	84	1885	1926	8	259	31.5	375	274	7.2	482	306	9.0	526
sessile / common Oak	Germany MUE	59/1	159	1901	1993	12	200	46.1	534	580	7.9	1095	701	10.4	1258
sessile / common Oak	Germany MUE	60/1	89	1900	1947	7	420	25.3	428	239	6.4	425	255	7.3	438
sessile / common Oak	Germany MUE	62/1	89	1900	1951	7	359	29.9	478	372	9.7	641	411	11.5	689
sessile / common Oak	Germany MUE	63/1	114	1900	1976	11	378	31.0	534	415	10.5	663	461	13.2	715
sessile / common Oak	Germany MUE	88/2	96	1934	1982	8	548	28.9	692	550	9.6	734	603	12.3	794
sessile / common Oak	Germany MUE	88/5	126	1934	2012	13	381	38.3	756	764	13.3	1101	888	17.2	1249
sessile / common Oak	Germany MUE	90/1	149	1934	2013	10	384	37.1	724	651	12.9	985	752	16.3	1107
sessile / common Oak	Poland	SCH_G/40	132	1900	1986	11	186	45.9	493	443	6.0	752	535	7.8	862
sessile / common Oak	Poland	SCH_G/41	147	1928	2001	10	181	47.5	506	502	9.0	833	611	12.0	997
sessile / common Oak	Poland	SCH_J/42	147	1900	1996	16	151	53.0	505	562	6.1	1031	700	8.0	1208
sessile / common Oak	Poland	SCH_J/43	167	1928	2016	16	169	51.6	541	604	7.7	1038	748	10.4	1261
sessile / common Oak	Poland	SCH_K/44	146	1928	2016	16	140	43.4	339	610	1.9	1045	728	3.2	1218
sessile / common Oak	Poland	SCH_K/45	146	1928	2016	16	250	43.4	606	610	9.5	1038	728	12.2	1218
sessile / common Oak	Poland	SCH_L/47	221	1900	2016	20	55	81.0	363	484	6.0	894	659	9.0	1177
sessile / common Oak	Poland	SCH_L/48	217	1929	2011	15	47	78.2	293	391	4.9	748	528	7.4	991
sessile / common Oak	Poland	SCH_M/49	141	1912	2016	18	130	51.6	416	386	7.5	701	478	10.5	836
sessile / common Oak	Poland	SCH_N/54	105	1907	1977	11	280	39.4	581	487	7.9	879	570	11.4	979
sessile / common Oak	Poland	SCH_N/55	71	1907	1943	7	840	20.1	592	302	9.5	541	307	10.4	539

sessile / common Oak	Poland	SCH_N/56	125	1931	1997	11	240	41.2	535	478	7.4	843	564	9.9	984
sessile / common Oak	Poland	SCH_N/57	120	1931	1992	10	184	44.9	471	432	7.7	715	519	10.3	845
Scots pine	Austria	308/40	55	1975	2014	10	1200	22.8	1035	577	20.8	943	283	10.0	464
Scots pine	Austria	308/50	55	1975	2014	10	1180	22.9	1025	581	20.1	920	284	9.7	452
Scots pine	England	Achnashellach/3254	59	1951	1982	9	410	35.5	719	349	13.6	592	168	6.5	286
Scots pine	England	Balmoral - Garmaddie Woods/3029	123	1920	2008	16	771	34.2	1272	779	25.4	1018	375	12.1	491
Scots pine	England	Black Isle - Findon/3245	54	1951	1983	9	693	24.1	653	276	15.5	458	135	7.5	224
Scots pine	England	Black Isle - Findon/3246	54	1951	1983	9	610	25.7	637	266	14.4	454	130	6.9	222
Scots pine	England	Black Isle - Findon/3247	50	1951	1979	8	699	23.6	638	250	9.4	426	122	4.5	209
Scots pine	England	Black Isle - Findon/3248	60	1951	1989	9	563	27.4	651	283	10.2	486	138	4.8	237
Scots pine	England	Black Isle - Findon/3249	45	1951	1974	6	707	23.8	653	253	13.9	418	123	6.7	205
Scots pine	England	Black Isle - Findon/3410	76	1963	2011	9	1168	23.4	1052	490	12.4	699	240	5.9	342
Scots pine	England	Black Isle - Findon/3411	76	1963	2011	9	1426	23.7	1312	563	11.9	643	275	5.7	315
Scots pine	England	Black Isle - Findon/3412	76	1963	2011	9	1191	26.6	1313	582	12.0	655	283	5.7	319
Scots pine	England	Black Isle - Findon/3413	76	1963	2011	9	843	31.7	1234	640	11.9	716	309	5.6	346
Scots pine	England	Brick Kiln/1104	137	1928	2002	16	334	51.8	1075	873	5.2	1303	413	2.4	618
Scots pine	England	Cannock Chase/1330	61	1952	1985	7	354	32.2	531	278	10.7	469	134	5.1	228
Scots pine	England	Cannock Chase/1332	78	1952	2003	10	544	32.9	844	526	12.2	680	254	5.7	329
Scots pine	England	Crowthorne/1435	50	1956	1976	5	803	19.7	549	185	11.0	301	91	5.3	149
Scots pine	England	Culbin/3138	60	1946	1982	11	610	25.4	624	243	6.9	403	118	3.3	197
Scots pine	England	Culbin/3139	88	1946	2009	15	980	26.0	1041	494	11.1	651	240	5.3	318
Scots pine	England	Cwmcarn/2093	38	1952	1972	6	610	21.9	494	149	14.2	228	73	6.9	112
Scots pine	England	Cwmcarn/2094	51	1952	1986	8	531	29.1	679	267	8.1	381	129	3.9	185
Scots pine	England	Cwmcarn/2095	51	1952	1986	8	610	27.5	710	291	9.8	419	141	4.7	204
Scots pine	England	Cwmcarn/2096	32	1952	1966	5	1102	17.2	607	136	9.6	208	67	4.6	103
Scots pine	England	Deer Park, Fochabers/3229	88	1950	2008	11	1205	27.6	1408	697	11.5	882	338	5.5	429
Scots pine	England	Deer Park, Fochabers/3230	62	1950	1982	8	768	25.9	813	359	10.3	606	175	4.9	297

Scots pine	England	Dilston - Dipton Wood/1076	131	1922	2001	14	266	48.6	772	575	21.3	940	273	9.9	448
Scots pine	England	Dilston - Dipton Wood/1077	131	1922	2001	14	658	37.7	1272	855	22.2	1059	410	10.6	509
Scots pine	England	Elveden-Brandon Park/1131	92	1938	1997	14	815	27.8	966	480	8.6	661	233	4.1	322
Scots pine	England	Glenbervie Inclosure/1429	37	1954	1973	6	1031	19.1	667	250	20.0	434	123	9.7	215
Scots pine	England	Glendye - Gallybank/3015	96	1920	1980	13	1051	27.0	1188	599	8.5	808	291	4.1	394
Scots pine	England	Glendye - Gallybank/3016	65	1920	1950	7	757	25.6	785	309	4.1	518	151	1.9	253
Scots pine	England	Laigh of Moray - Roseisle/3344	31	1949	1966	6	1348	17.3	745	209	17.3	343	103	8.4	170
Scots pine	England	Laigh of Moray - Roseisle/3345	37	1949	1971	7	608	25.8	640	221	15.5	344	108	7.4	168
Scots pine	England	Laigh of Moray - Roseisle/3347	37	1949	1971	7	814	22.8	702	238	18.1	389	116	8.8	191
Scots pine	England	Novar - Evanton Wood/3033	78	1921	1971	13	941	30.0	1260	768	18.2	1049	372	8.7	510
Scots pine	England	Rendlesham/1153	38	1941	1959	8	652	21.9	526	180	11.3	298	88	5.4	147
Scots pine	England	Seafield - Achvochie/3064	89	1924	1970	10	1542	22.8	1334	497	5.4	659	243	2.6	323
Scots pine	England	Seafield Estate - Curr Wood/3069	102	1925	1982	12	1203	27.5	1399	672	11.9	871	327	5.7	424
Scots pine	England	South Oakley Inclosure/1313	88	1952	2013	14	794	31.9	1175	690	3.3	874	333	1.5	423
Scots pine	England	South Oakley/1312	77	1952	2003	12	365	40.4	788	507	32.8	810	242	15.6	390
Scots pine	England	Swaffham/1133	91	1938	2012	17	758	31.2	1079	630	5.5	895	304	2.6	434
Scots pine	England	Swaffham/1134	40	1938	1961	9	675	22.2	559	196	12.8	344	96	6.2	169
Scots pine	Germany BW	Kie 120/3	54	1981	2012	7	1037	19.2	679	237	8.2	383	117	4.0	189
Scots pine	Germany BW	Kie 156/2	83	1956	2015	17	575	31.0	812	564	7.5	915	273	3.5	444
Scots pine	Germany BW	Kie 157/2	81	1956	2015	17	593	31.1	842	587	7.1	887	284	3.4	430
Scots pine	Germany BW	Kie 158/2	81	1956	2015	17	600	31.9	887	648	8.5	1000	313	4.0	484
Scots pine	Germany GOE	ID 462711/2	57	1973	2001	6	915	22.7	784	331	10.7	499	162	5.1	245
Scots pine	Germany GOE	ID 911711/6	39	1991	2014	6	2084	16.9	1112	400	20.0	460	198	9.7	228
Scots pine	Germany GOE	ID 911711/8	39	1991	2014	6	2251	15.7	1067	344	17.0	384	171	8.3	191
Scots pine	Germany GOE	ID 912711/7	38	1991	2014	6	2136	15.4	981	318	13.0	359	158	6.3	179
Scots pine	Germany GOE	ID 913711/7	39	1991	2014	6	1924	16.3	968	316	17.2	356	157	8.4	177
Scots pine	Germany GOE	ID H09711/1	100	1934	1993	9	508	31.0	717	450	7.7	761	218	3.7	369

Scots pine	Germany GOE	ID H09711/3	94	1941	1997	9	704	26.2	759	357	8.0	591	174	3.7	289
Scots pine	Germany GOE	ID H13711/1	121	1890	1986	16	402	36.2	728	524	5.4	877	252	2.5	423
Scots pine	Germany GOE	ID H13711/2	141	1890	2006	19	352	40.3	757	607	10.7	977	290	5.0	470
Scots pine	Germany GOE	ID H14711/1	132	1892	1986	16	460	36.2	833	614	1.8	981	295	0.8	474
Scots pine	Germany GOE	ID H14711/2	100	1892	1954	12	516	32.3	778	456	7.1	765	220	3.4	371
Scots pine	Germany GOE	ID H15711/2	155	1926	2006	11	340	39.0	694	484	3.9	810	232	1.8	389
Scots pine	Germany GOE	ID S04711/3	62	1963	2011	11	897	25.0	897	483	13.4	659	235	6.4	322
Scots pine	Germany MUE	209/12	124	1952	2013	10	348	34.5	584	393	7.5	538	189	3.5	260
Scots pine	Germany MUE	209/13	124	1952	2013	10	565	27.6	662	389	6.9	495	189	3.3	241
Scots pine	Germany MUE	225/6	93	1959	2012	10	809	32.1	1208	574	12.8	923	277	6.1	448
Scots pine	Germany MUE	226/4	79	1959	1996	8	510	27.4	591	334	11.8	598	162	5.6	293
Scots pine	Germany MUE	228/1	106	1959	2013	10	453	28.7	565	333	9.8	567	161	4.7	277
Scots pine	Germany MUE	229/12	81	1961	2006	9	550	26.6	608	340	8.4	559	165	4.0	273
Scots pine	Germany MUE	230/4	92	1962	2009	10	520	28.3	634	349	6.6	524	169	3.1	256
Scots pine	Germany MUE	234/11	129	1962	2006	10	367	27.8	435	230	6.7	492	112	3.2	241
Scots pine	Germany MUE	238/5	98	1967	2005	8	583	24.6	568	281	8.5	525	137	4.1	257
Scots pine	Germany MUE	52/2	131	1899	1995	13	383	43.3	925	633	9.3	1314	302	4.4	632
Scots pine	Germany MUE	53/2	154	1899	2014	15	294	41.1	653	604	9.8	1084	289	4.6	523
Scots pine	Germany MUE	57/1	113	1901	1970	11	672	23.2	596	257	6.0	406	126	2.9	199
Scots pine	Germany MUE	58/2	148	1901	2014	15	300	34.7	508	331	5.2	733	159	2.5	356
Scots pine	Germany MUE	610/14	62	1976	2014	7	1361	21.1	1037	483	14.7	574	237	7.1	282
Scots pine	Germany MUE	610/2	62	1976	2014	7	1157	20.1	815	338	8.7	430	166	4.2	212
Scots pine	Germany MUE	79/2	122	1912	2008	12	452	35.1	779	565	8.8	876	272	4.2	424
Scots pine	Poland	Lipce/1	50	1975	2014	11	1157	21.0	874	412	17.3	505	202	8.4	249
Scots pine	Poland	Lipce/2	50	1975	2014	11	1161	20.2	827	380	15.1	472	187	7.4	233
Scots pine	Poland	Lipce/3	50	1975	2014	11	1031	22.2	853	419	18.8	521	205	9.1	256
Scots pine	Poland	Lipce/4	50	1975	2014	11	1086	21.6	856	411	18.0	508	202	8.8	250
Scots pine	Poland	Lipce/5	50	1975	2014	11	973	22.3	808	398	18.5	525	195	9.0	258
Scots pine	Poland	SCH_A/1	102	1911	1978	10	408	33.8	662	452	4.2	731	218	2.0	354

Scots pine	Poland	SCH_A/2	66	1911	1942	5	635	25.0	635	313	7.8	452	153	3.7	221
Scots pine	Poland	SCH_A/3	66	1911	1942	5	473	26.8	529	266	7.2	422	129	3.4	206
Scots pine	Poland	SCH_A/4	66	1911	1942	5	750	23.2	665	322	8.3	449	157	4.0	220
Scots pine	Poland	SCH_A/5	66	1911	1942	5	438	26.9	493	260	7.3	430	126	3.5	210
Scots pine	Poland	SCH_A/6	92	1911	1968	8	333	34.9	569	374	8.4	594	180	4.0	287
Scots pine	Poland	SCH_A/8	129	1928	2013	16	130	49.7	393	622	10.8	968	295	5.0	461
Scots pine	Poland	SCH_B/14	137	1928	2013	16	268	46.1	716	688	8.2	1059	327	3.8	505
Scots pine	Poland	SCH_B/15	137	1911	2013	17	188	51.9	607	598	8.2	1019	283	3.8	485
Scots pine	Poland	SCH_B/16	107	1911	1983	11	204	44.8	520	449	3.4	742	214	1.6	355
Scots pine	Poland	SCH_B/17	107	1928	1983	10	283	37.4	541	446	4.6	761	214	2.2	366
Scots pine	Poland	SCH_B/18	102	1911	1978	10	204	40.9	450	353	3.4	577	169	1.6	277
Scots pine	Poland	SCH_B/19	102	1911	1978	10	204	40.9	450	353	2.6	565	169	1.2	272
Scots pine	Poland	SCH_C/23	137	1932	2013	15	277	41.8	633	564	6.4	927	269	3.0	445
Scots pine	Poland	SCH_D/27	137	1928	2015	16	238	47.8	674	634	9.0	995	301	4.2	475
Scots pine	Poland	SCH_D/28	137	1928	2015	16	316	43.8	778	724	9.0	1144	345	4.2	548
Scots pine	Spain	AV-1/1	135	1963	2015	8	340	48.2	975	827	7.9	1239	393	3.7	589
Scots pine	Spain	BU-1/1	139	1963	2015	8	719	30.7	1000	524	3.7	647	253	1.8	313
Scots pine	Spain	BU-3/1	110	1963	2015	8	640	35.1	1103	704	6.1	886	339	2.9	427
Scots pine	Spain	BU-7/1	138	1963	2015	8	940	28.8	1180	582	1.7	784	282	0.8	380
Scots pine	Spain	COV/1A	95	1968	2013	9	1142	22.2	944	333	4.5	394	163	2.2	193
Scots pine	Spain	COV/1C	95	1968	2013	9	943	23.4	848	294	3.8	389	144	1.9	191
Scots pine	Spain	COV/2A	95	1968	2013	9	1413	21.4	1101	404	6.2	495	198	3.0	243
Scots pine	Spain	COV/2C	95	1968	2013	9	840	25.8	884	344	5.1	462	167	2.5	225
Scots pine	Spain	COV/3A	95	1968	2013	9	1152	24.1	1086	397	5.2	468	194	2.5	229
Scots pine	Spain	DUR/1A	86	1968	2013	9	1217	23.5	1102	414	7.4	539	202	3.5	264
Scots pine	Spain	DUR/2A	86	1968	2013	9	1267	23.6	1155	434	4.5	560	212	2.1	274
Scots pine	Spain	DUR/2C	86	1968	2013	9	883	26.9	993	380	5.8	522	185	2.8	254
Scots pine	Spain	DUR/3A	86	1968	2013	9	1567	21.4	1221	391	4.7	445	192	2.2	218
Scots pine	Spain	DUR/3C	86	1968	2013	9	821	27.1	934	366	4.9	515	178	2.3	251

Scots pine	Spain	GU-5/1	129	1964	2015	7	496	35.2	859	506	3.4	596	243	1.6	287
Scots pine	Spain	GU-6/1	108	1964	2015	7	1547	22.4	1297	451	3.6	483	221	1.7	237
Scots pine	Spain	GU-8/1	86	1964	2014	7	1552	22.0	1264	599	6.4	802	293	3.0	394
Scots pine	Spain	GU-9/1	110	1964	2015	7	823	27.7	970	509	4.1	682	247	2.0	331
Scots pine	Spain	M-1/1	93	1963	2015	8	487	44.1	1211	857	10.4	1046	408	4.9	500
Scots pine	Spain	M-2/1	94	1963	2015	8	780	35.9	1394	904	13.0	1163	434	6.1	560
Scots pine	Spain	M-3/1	100	1963	2015	8	390	39.7	819	507	4.5	551	243	2.1	264
Scots pine	Spain	M-4/1	99	1963	2015	8	545	31.7	798	384	2.9	408	185	1.4	197
Scots pine	Spain	NEILA/1A	81	1972	2012	8	1114	27.6	1306	713	16.5	920	346	7.9	448
Scots pine	Spain	NEILA/2A	81	1972	2012	8	1580	25.1	1590	807	16.9	961	393	8.1	469
Scots pine	Spain	NEILA/3A	81	1972	2012	8	1230	26.5	1351	768	16.9	967	373	8.1	471
Scots pine	Spain	ROBLE/1A	52	1982	2012	7	1550	23.9	1442	519	15.6	528	253	7.5	258
Scots pine	Spain	ROBLE/2A	52	1982	2012	7	1370	25.2	1388	525	15.3	554	256	7.4	270
Scots pine	Spain	ROBLE/3A	52	1982	2012	7	1300	24.9	1292	505	13.6	522	246	6.6	254
Scots pine	Spain	SO-1/1	143	1963	2015	8	596	35.1	1027	676	3.7	822	325	1.8	395
Scots pine	Spain	SO-24/1	88	1963	2015	8	1200	27.1	1366	651	8.5	813	316	4.1	396
Scots pine	Spain	SO-3/1	93	1963	2015	8	1170	24.7	1148	462	5.7	576	225	2.8	282
Scots pine	Spain	SO_18/1	116	1963	2015	8	550	34.6	927	484	5.2	574	233	2.4	277
Scots pine	Spain	SO_8/1	100	1963	2015	8	900	29.3	1161	606	4.9	760	294	2.3	369
Scots pine	Spain	TE-10/1	93	1964	2015	8	1270	23.6	1158	456	6.2	550	223	3.0	269
Scots pine	Spain	TE-14/1	115	1964	2015	8	808	27.4	936	375	2.8	442	182	1.3	215
Scots pine	Spain	TE-15/1	118	1964	2015	8	536	36.6	988	569	5.6	676	273	2.7	325
Scots pine	Spain	TE-16/1	124	1964	2015	8	981	27.4	1136	445	4.5	493	216	2.2	240
Scots pine	Spain	TE-3/1	111	1964	2015	8	767	30.9	1078	477	4.8	510	231	2.3	246
Scots pine	Sweden	1000/5	62	1981	2007	5	1750	17.1	947	375	8.6	396	185	4.2	196
Scots pine	Sweden	1004/4	77	1975	2006	5	978	20.5	713	316	8.1	435	155	3.9	214
Scots pine	Sweden	1005/4	72	1980	2015	7	710	23.9	662	340	8.6	472	166	4.2	231
Scots pine	Sweden	1005/6	72	1980	2015	7	1690	18.4	1034	468	10.9	497	231	5.3	245
Scots pine	Sweden	1006/1	66	1978	2014	6	710	21.5	558	247	7.2	383	121	3.5	188

Scots pine	Sweden	1006/6	66	1978	2014	6	2120	16.1	1044	419	9.2	450	208	4.5	224
Scots pine	Sweden	1007/2	77	1977	2015	6	960	21.3	745	349	6.4	431	171	3.1	212
Scots pine	Sweden	1008/2	67	1976	2015	6	1490	17.8	863	347	8.0	377	172	3.9	187
Scots pine	Sweden	1008/3	67	1976	2015	6	2170	16.4	1099	416	7.8	449	206	3.8	223
Scots pine	Sweden	1008/4	67	1986	2015	4	580	22.6	493	212	5.5	323	104	2.7	159
Scots pine	Sweden	1009/4	71	1981	2015	6	1010	20.1	709	299	5.7	326	147	2.7	161
Scots pine	Sweden	1009/6	71	1981	2015	6	510	23.0	446	202	5.4	291	99	2.6	143
Scots pine	Sweden	895/12	76	1970	2011	7	1610	19.0	1035	485	12.3	532	239	6.0	263
Scots pine	Sweden	895/2	70	1970	2005	6	470	24.5	456	221	5.3	364	108	2.5	178
Scots pine	Sweden	900/4	77	1966	2007	7	650	26.0	691	360	5.8	529	175	2.7	258
Scots pine	Sweden	902/1	75	1969	2007	6	750	23.6	686	318	4.1	388	156	1.9	190
Scots pine	Sweden	910/3	77	1966	2006	6	838	21.8	674	282	5.0	316	138	2.4	155
Scots pine	Sweden	912/1	87	1969	2016	6	1055	21.6	831	399	8.8	480	196	4.2	235
Scots pine	Sweden	913/4	82	1972	2006	6	1846	15.8	881	295	8.2	319	147	4.0	158
Scots pine	Sweden	918/4	84	1970	2006	6	1230	19.8	843	363	11.0	446	179	5.2	220
Scots pine	Sweden	922/9	86	1967	2007	7	1130	21.6	896	430	9.9	512	211	4.8	252
Scots pine	Sweden	923/1	75	1973	2007	5	1190	20.4	856	360	7.1	476	177	3.4	234
Scots pine	Sweden	924/9	59	1968	1993	5	830	21.0	625	265	7.7	350	130	3.7	172
Scots pine	Sweden	926/8	95	1969	2014	6	980	22.6	833	401	5.6	465	196	2.7	228
Scots pine	Sweden	927/6	83	1969	2007	7	870	20.6	638	277	3.5	377	136	1.7	186
Scots pine	Sweden	927/9	83	1969	2007	7	1190	19.4	795	354	5.9	437	174	2.9	215
Scots pine	Sweden	929/2	91	1970	2016	8	970	24.2	920	458	9.2	559	223	4.4	273
Scots pine	Sweden	930/3	73	1966	2007	7	640	24.9	635	327	6.9	481	159	3.3	235
Scots pine	Sweden	933/4	92	1973	2015	6	710	22.5	599	242	3.4	287	118	1.6	141
Scots pine	Sweden	935/3	73	1971	2007	7	1050	21.9	850	398	9.7	457	195	4.7	224
Scots pine	Sweden	936/3	69	1974	2007	6	1920	16.4	973	339	6.7	356	168	3.3	177
Scots pine	Sweden	938/6	89	1974	2005	5	460	19.8	317	116	4.0	195	57	2.0	97
Scots pine	Sweden	938/7	89	1974	2005	5	1860	14.4	770	240	5.7	245	120	2.8	122
Scots pine	Sweden	939/2	61	1981	2006	5	800	21.1	608	257	9.4	392	126	4.6	193

Scots pine	Sweden	939/4	61	1981	2006	5	1820	17.7	1042	419	10.9	452	207	5.3	224
Scots pine	Sweden	940/9	92	1968	2013	7	667	25.7	696	353	4.8	390	172	2.3	190
Scots pine	Sweden	945/6	64	1973	2014	8	1040	21.6	819	393	12.0	573	193	5.8	282
Scots pine	Sweden	945/8	64	1973	2014	9	1650	18.9	1053	511	19.1	638	252	9.3	315
Scots pine	Sweden	946/2	73	1976	2015	7	1580	18.3	954	433	13.2	496	214	6.4	245
Scots pine	Sweden	947/1	73	1978	2015	6	1250	19.2	817	360	9.8	441	178	4.7	218
Scots pine	Sweden	947/4	73	1978	2015	6	800	22.0	654	305	10.1	412	149	4.9	203
Scots pine	Sweden	948/1	75	1975	2015	7	620	26.0	660	347	9.2	484	169	4.4	237
Scots pine	Sweden	948/5	75	1975	2015	7	1023	22.1	841	431	9.8	556	211	4.7	273
Scots pine	Sweden	951/3	89	1975	2014	6	1500	18.1	892	342	5.2	396	169	2.5	196
Scots pine	Sweden	952/2	94	1975	2014	7	1070	18.6	668	285	5.4	377	141	2.6	186
Scots pine	Sweden	989/5	86	1972	2006	6	1040	22.1	853	393	7.1	497	192	3.4	244
Scots pine	Sweden	990/2	86	1972	2006	6	660	22.3	550	229	5.1	356	112	2.5	175
Scots pine	Sweden	990/6	86	1972	2006	6	1460	17.6	832	296	4.4	383	146	2.1	190
Scots pine	Sweden	991/1	70	1976	2006	5	1210	17.1	660	247	7.0	355	122	3.4	176
Scots pine	Sweden	993/1	84	1983	2014	5	740	19.3	489	181	7.4	236	89	3.6	117
Scots pine	Sweden	993/3	84	1983	2014	5	1330	16.8	701	239	7.7	241	118	3.8	119
Scots pine	Sweden	994/1	88	1983	2014	5	1290	17.1	698	245	5.4	287	121	2.6	142
Scots pine	Sweden	994/2	88	1983	2014	5	530	21.3	410	154	3.6	208	76	1.8	102
Scots pine	Sweden	996/7	70	1973	2015	7	1448	21.3	1121	585	12.5	620	287	6.1	304
Scots pine	Sweden	999/1	74	1976	2014	6	1310	21.0	989	500	11.5	544	246	5.6	267
Norway spruce	Austria	220/10	75	1968	2003	8	1070	27.0	1211	761	19.0	982	393	9.7	507
Norway spruce	Austria	220/12	75	1968	2003	8	1040	26.7	1156	693	16.8	872	358	8.6	451
Norway spruce	Austria	220/20	75	1968	2003	8	1470	23.2	1304	701	18.0	874	364	9.2	454
Norway spruce	Austria	220/22	75	1968	2003	8	1240	26.1	1329	820	23.4	1010	424	12.0	523
Norway spruce	Austria	220/30	75	1968	2003	8	1660	22.5	1402	759	22.0	894	394	11.3	465
Norway spruce	Austria	220/32	75	1968	2003	8	1230	25.6	1278	751	23.8	920	388	12.2	476
Norway spruce	Austria	301/10	67	1977	2014	9	1262	24.2	1198	748	26.3	1099	387	13.3	571
Norway spruce	Austria	301/12	67	1974	2014	10	567	30.0	760	518	19.4	788	266	9.8	408

Norway spruce	Austria	301/20	67	1977	2014	9	976	24.9	970	616	23.5	884	319	11.9	459
Norway spruce	Austria	301/22	67	1974	2014	10	550	33.1	863	650	26.0	938	333	13.2	484
Norway spruce	Austria	302/10	52	1982	2002	5	1935	18.5	1193	510	26.8	762	267	13.7	399
Norway spruce	Austria	302/20	52	1982	2002	5	1845	18.6	1148	431	17.6	600	225	9.0	314
Norway spruce	Austria	303/30	52	1977	2002	6	2075	16.5	1065	383	15.4	518	201	7.9	272
Norway spruce	Austria	303/40	52	1977	2002	6	1627	19.4	1083	456	18.4	629	238	9.4	329
Norway spruce	Austria	303/50	52	1977	2002	6	1675	18.7	1051	416	15.8	621	217	8.0	325
Norway spruce	Austria	304/1	41	1969	1997	8	2390	17.1	1299	576	24.0	768	302	12.4	403
Norway spruce	Austria	304/10	41	1969	1997	7	2000	16.7	1047	459	22.5	701	241	11.5	368
Norway spruce	Austria	304/11	37	1969	1993	6	1020	22.2	843	407	28.8	674	211	14.8	351
Norway spruce	Austria	304/16	37	1969	1993	6	2030	18.3	1230	538	29.6	585	281	15.3	306
Norway spruce	Austria	304/2	37	1969	1993	6	1020	22.1	837	403	27.2	652	209	14.0	340
Norway spruce	Austria	304/7	37	1969	1993	6	1330	19.1	863	396	33.8	669	207	17.5	349
Norway spruce	Denmark	IS/A	75	1936	1967	12	811	19.5	544	225	18.3	379	117	9.4	199
Norway spruce	Denmark	IS/C	62	1935	1954	12	1071	15.4	490	141	11.2	234	74	5.8	123
Norway spruce	Denmark	IS/D	78	1936	1970	14	903	18.7	569	230	12.4	407	120	6.4	214
Norway spruce	Denmark	IS/F	62	1935	1954	12	1007	16.1	499	157	12.9	249	82	6.7	131
Norway spruce	Denmark	IS/H	75	1936	1967	12	840	19.7	575	235	16.8	408	123	8.6	214
Norway spruce	Denmark	IS/I	75	1934	1967	13	995	17.4	558	201	13.9	335	105	7.2	177
Norway spruce	Denmark	IS/K	91	1936	1983	18	1086	19.6	734	330	8.5	543	172	4.4	285
Norway spruce	Denmark	IS/L	66	1934	1958	11	1781	13.8	685	195	9.3	309	103	4.8	164
Norway spruce	Denmark	IS/M	91	1936	1983	20	1933	15.2	874	319	3.0	490	168	1.5	258
Norway spruce	Denmark	IS/N	78	1934	1970	14	626	20.7	463	201	13.9	353	105	7.1	185
Norway spruce	Denmark	IS/O	78	1936	1970	14	937	18.7	586	236	13.5	416	124	6.9	219
Norway spruce	Denmark	IS/Q	78	1936	1970	13	1539	16.6	796	289	11.0	441	152	5.7	232
Norway spruce	Denmark	KM/1	53	1964	2000	10	1758	19.7	1201	599	22.1	841	312	11.3	439
Norway spruce	Denmark	KM/2	41	1964	1988	8	1772	18.2	1063	493	27.8	683	258	14.3	358
Norway spruce	Denmark	KM/5	41	1964	1988	13	729	25.8	769	439	30.4	755	227	15.5	393
Norway spruce	Denmark	KM/6	29	1964	1976	6	2034	13.6	765	225	18.4	377	119	9.5	200

Norway spruce	England	Achnashellach/3299	70	1957	1994	9	381	46.5	1030	756	14.2	1271	383	7.1	647
Norway spruce	England	Bennan/3164	75	1947	2000	13	824	35.2	1424	1059	16.3	1385	541	8.2	710
Norway spruce	England	Bowmont/3651	72	1930	1983	12	1877	24.0	1761	809	12.7	1018	419	6.5	528
Norway spruce	England	Bowmont/3652	64	1930	1975	11	1951	22.3	1618	677	16.3	852	352	8.1	443
Norway spruce	England	Bowmont/3653	64	1930	1975	11	1802	22.5	1523	649	22.6	845	337	11.3	439
Norway spruce	England	Bowmont/3654	72	1930	1983	12	1951	23.7	1787	810	12.7	1004	420	6.5	521
Norway spruce	England	Bowmont/3661	64	1930	1975	11	1037	27.4	1203	610	11.5	935	314	5.6	484
Norway spruce	England	Bowmont/3662	62	1930	1974	10	988	28.8	1239	607	17.0	1019	312	8.6	527
Norway spruce	England	Bowmont/3663	64	1930	1975	11	963	28.5	1190	595	5.4	997	306	2.6	516
Norway spruce	England	Bowmont/3664	72	1930	1983	12	1012	30.9	1423	708	12.8	1107	363	6.4	572
Norway spruce	England	Clocaenog/2098	61	1954	1996	10	1651	23.1	1452	609	9.7	759	316	4.9	394
Norway spruce	England	Clocaenog/2099	61	1954	1996	10	816	30.0	1090	586	18.2	913	301	9.2	471
Norway spruce	England	Clunes/3426	79	1968	2013	10	747	35.1	1290	1173	60.3	1647	599	30.6	843
Norway spruce	England	Clunes/3427	79	1968	2013	10	914	36.4	1673	1347	33.2	1645	687	16.8	841
Norway spruce	England	Clunes/3428	79	1968	2013	10	820	37.5	1571	1319	36.2	1595	673	18.2	814
Norway spruce	England	Coed-y-Brenin/2046	53	1948	1978	8	419	43.7	1028	743	17.0	1189	377	8.5	606
Norway spruce	England	Coed-y-Brenin_2/2048	52	1948	1978	8	735	31.0	1037	555	25.1	826	285	12.7	426
Norway spruce	England	Dodd Wood/1266	37	1950	1962	5	2022	16.2	1012	226	13.4	367	119	6.9	193
Norway spruce	England	Drumlanrig/3006	75	1919	1967	10	1039	30.9	1458	842	10.6	1060	432	5.3	545
Norway spruce	England	Glenbranter/3218	44	1949	1971	8	546	30.3	744	379	21.1	630	195	10.7	326
Norway spruce	England	Glentress/3142	76	1946	1977	9	412	37.7	795	547	1.8	900	279	0.8	460
Norway spruce	England	Rheola/2158	77	1958	2010	11	981	35.2	1695	1158	44.3	1500	592	22.3	769
Norway spruce	England	Rheola/2159	77	1958	2010	11	508	44.7	1290	901	16.0	1500	456	7.9	764
Norway spruce	England	Rheola/2160	44	1958	1977	5	618	32.9	961	454	21.2	655	232	10.8	337
Norway spruce	England	Soudley - Abbotswood/1090	70	1927	1973	10	437	37.4	833	642	31.0	1022	328	15.6	524
Norway spruce	England	Tintern/2015	84	1928	1984	13	348	48.6	1011	908	60.5	1522	459	29.9	774
Norway spruce	England	Tintern/2016	101	1928	2002	16	509	43.0	1214	1039	14.3	1588	527	7.1	809
Norway spruce	Germany BW	Fi 18/1	75	1873	1918	8	888	28.6	1102	755	24.7	1054	389	12.6	544
Norway spruce	Germany BW	Fi 26/A	57	1873	1900	7	956	25.3	974	574	19.4	845	297	9.7	437

Norway spruce	Germany BW	Fi 27/1	83	1873	1926	12	562	35.9	1005	861	24.5	1439	439	12.2	738
Norway spruce	Germany BW	Fi 28/1	75	1873	1918	10	528	35.8	940	759	20.9	1356	388	10.4	696
Norway spruce	Germany BW	Fi 57/1	71	1874	1913	7	1512	20.4	1091	625	23.7	788	326	12.2	411
Norway spruce	Germany BW	Fi 58/1	64	1874	1908	6	1444	18.7	906	435	14.8	539	227	7.7	282
Norway spruce	Germany BW	Fi 81/1	127	1874	1957	13	544	33.2	858	672	9.7	1097	344	4.9	564
Norway spruce	Germany BW	Fi 82/1	81	1887	1929	7	1480	20.2	1051	518	16.3	713	270	8.4	372
Norway spruce	Germany BW	Fi 113/1	191	1881	1996	15	540	31.9	799	497	7.8	748	255	3.9	385
Norway spruce	Germany BW	Fi 151/1	90	1886	1934	9	760	27.3	875	563	16.4	893	291	8.3	462
Norway spruce	Germany BW	Fi 154/1	77	1887	1934	9	790	29.3	1019	745	21.0	1141	383	10.6	589
Norway spruce	Germany BW	Fi 155/1	59	1888	1919	6	1210	22.4	1014	555	17.6	798	288	8.9	415
Norway spruce	Germany BW	Fi 157/1	71	1889	1935	8	756	31.7	1107	871	22.5	1266	447	11.3	652
Norway spruce	Germany BW	Fi 158/1	71	1889	1935	8	632	31.3	907	667	17.8	1151	342	8.9	593
Norway spruce	Germany BW	Fi 162/1	70	1892	1935	8	756	30.0	1013	749	17.8	1156	385	9.0	597
Norway spruce	Germany BW	Fi 167/1	71	1893	1931	8	948	23.9	882	515	14.7	787	267	7.5	409
Norway spruce	Germany BW	Fi 169/1	69	1893	1929	7	1025	23.0	897	511	18.8	766	265	9.6	399
Norway spruce	Germany BW	Fi 170/1	71	1895	1937	7	1088	23.0	952	553	19.3	856	287	9.9	445
Norway spruce	Germany BW	Fi 171/1	70	1895	1937	7	648	28.6	804	547	19.1	965	282	9.6	499
Norway spruce	Germany BW	Fi 192/1	73	1903	1940	8	748	27.4	867	586	15.3	946	302	7.8	489
Norway spruce	Germany BW	Fi 193/1	77	1903	1940	8	535	30.3	728	519	13.6	858	267	6.8	443
Norway spruce	Germany BW	Fi 194/1	72	1903	1934	7	730	26.3	792	497	16.9	845	257	8.6	438
Norway spruce	Germany BW	Fi 195/1	76	1903	1940	8	710	25.3	724	451	14.2	690	233	7.1	358
Norway spruce	Germany BW	Fi 196/1	86	1913	1964	10	810	27.0	916	617	22.5	892	318	11.5	462
Norway spruce	Germany BW	Fi 197/1	69	1903	1934	7	808	22.4	677	336	10.7	500	174	5.5	260
Norway spruce	Germany BW	Fi 198/1	70	1903	1946	9	752	26.5	826	498	12.3	860	257	6.2	446
Norway spruce	Germany BW	Fi 199/1	128	1913	2013	20	360	43.8	885	868	10.8	1457	440	5.3	743
Norway spruce	Germany BW	Fi 200/1	91	1913	1976	15	540	31.4	779	551	13.3	930	283	6.7	480
Norway spruce	Germany BW	Fi 201/1	76	1913	1961	10	688	28.3	839	566	16.5	869	292	8.3	449
Norway spruce	Germany BW	Fi 204/A	56	1913	1939	6	1848	15.4	849	272	14.4	388	143	7.5	205
Norway spruce	Germany BW	Fi 205/1	105	1913	1988	15	632	34.9	1080	860	16.0	1323	439	8.1	680

Norway spruce	Germany BW	Fi 206/1	91	1913	1976	14	648	30.3	882	626	22.3	1115	322	11.2	576
Norway spruce	Germany BW	Fi 207/1	100	1913	1988	15	528	35.3	919	723	17.8	1232	369	9.0	633
Norway spruce	Germany BW	Fi 208/1	88	1913	1976	14	640	30.2	867	605	19.8	1031	311	10.0	532
Norway spruce	Germany BW	Fi 215/1	71	1904	1943	8	748	29.3	965	662	21.2	1071	341	10.7	553
Norway spruce	Germany BW	Fi 216/1	64	1904	1936	7	856	26.2	923	567	17.5	889	293	8.8	461
Norway spruce	Germany BW	Fi 217/1	71	1904	1943	8	832	27.1	947	612	21.8	960	316	11.0	498
Norway spruce	Germany BW	Fi 218/1	71	1904	1943	8	676	29.6	886	599	16.7	994	308	8.4	514
Norway spruce	Germany BW	Fi 219/1	64	1904	1936	7	868	26.2	936	596	20.4	927	308	10.3	480
Norway spruce	Germany BW	Fi 220/1	64	1904	1936	7	724	27.0	819	531	15.4	963	274	7.7	499
Norway spruce	Germany BW	Fi 221/1	71	1904	1943	8	672	29.1	857	565	17.5	998	291	8.8	516
Norway spruce	Germany BW	Fi 222/1	76	1904	1948	9	636	27.6	745	500	10.0	884	258	5.0	458
Norway spruce	Germany BW	Fi 223/A	85	1905	1958	8	720	29.0	914	628	13.4	977	323	6.8	505
Norway spruce	Germany BW	Fi 235/1	64	1905	1938	7	712	28.3	869	575	18.7	957	296	9.4	494
Norway spruce	Germany BW	Fi 236/1	65	1905	1938	7	1028	21.5	807	383	14.6	623	199	7.5	325
Norway spruce	Germany BW	Fi 244/1	72	1907	1948	9	550	31.8	809	607	17.3	1068	311	8.7	550
Norway spruce	Germany BW	Fi 261/1	60	1924	1961	9	848	25.8	892	577	25.6	972	298	13.0	504
Norway spruce	Germany BW	Fi 262/1	80	1913	1966	12	668	28.5	824	573	16.2	916	295	8.2	474
Norway spruce	Germany BW	Fi 263/1	80	1919	1966	11	580	31.7	849	640	19.4	1005	328	9.8	518
Norway spruce	Germany BW	Fi 280/1	83	1935	1997	14	416	42.9	990	953	20.9	1592	484	10.4	813
Norway spruce	Germany BW	Fi 281/1	56	1935	1968	9	1272	24.5	1231	798	25.1	1048	413	12.7	543
Norway spruce	Germany BW	Fi 386/10	40	1983	2002	7	1250	21.3	967	509	29.1	676	265	14.9	352
Norway spruce	Germany BW	Fi 386/12	44	1983	2002	7	1600	21.8	1284	722	34.4	806	375	17.6	419
Norway spruce	Germany BW	Fi 386/4	40	1983	2002	7	1110	18.7	697	299	21.6	512	156	11.1	268
Norway spruce	Germany BW	Fi 393/3	75	1958	1988	7	662	27.9	790	548	18.6	960	282	9.4	496
Norway spruce	Germany BW	Fi 399/2	69	1980	2015	8	1116	28.5	1377	862	31.6	965	444	16.1	498
Norway spruce	Germany BW	Fi 401/2D	70	1969	2014	13	520	39.2	1070	958	30.7	1348	488	15.4	689
Norway spruce	Germany BW	Fi 405/10	55	1982	2015	10	1540	24.5	1491	1027	34.5	1186	531	17.6	614
Norway spruce	Germany BW	Fi 405/4	55	1982	2015	10	1710	23.5	1548	1007	35.6	1134	522	18.2	588
Norway spruce	Germany BW	Fi 469/1	76	1879	1918	6	652	27.6	764	473	11.4	835	244	5.8	432

Norway spruce	Germany GOE	ID 054511/2	137	1916	2014	21	471	42.5	1104	1021	12.2	1506	518	6.0	768
Norway spruce	Germany GOE	ID 064511/2	89	1928	1994	18	463	39.1	949	790	14.0	1260	402	6.9	644
Norway spruce	Germany GOE	ID 064511/3	84	1933	1989	16	392	40.5	850	711	15.4	1140	362	7.8	582
Norway spruce	Germany GOE	ID 071511/2	118	1928	2004	18	535	42.5	1254	1234	14.2	1707	626	7.0	870
Norway spruce	Germany GOE	ID 075511/3	103	1928	1994	18	432	38.3	857	759	10.6	1284	387	5.3	657
Norway spruce	Germany GOE	ID 075511/5	79	1928	1970	11	616	31.9	911	659	12.8	982	338	6.4	505
Norway spruce	Germany GOE	ID 103511/4	132	1938	2009	16	340	45.3	883	852	18.6	1378	432	9.3	701
Norway spruce	Germany GOE	ID 103511/5	137	1938	2014	17	332	45.2	859	786	9.2	1310	398	4.5	667
Norway spruce	Germany GOE	ID 116511/0	129	1951	2012	14	276	47.0	760	696	4.0	1006	352	2.0	510
Norway spruce	Germany GOE	ID 471511/4	57	1978	2012	9	470	35.6	829	612	21.8	984	313	10.9	504
Norway spruce	Germany GOE	ID 474511/2	57	1978	2012	8	530	31.8	780	484	21.4	748	248	10.8	385
Norway spruce	Germany GOE	ID 474511/4	57	1978	2012	8	1415	23.6	1290	676	23.0	762	350	11.7	395
Norway spruce	Germany GOE	ID 555511/3	56	1988	2012	6	780	27.1	888	462	19.6	630	238	10.0	325
Norway spruce	Germany GOE	ID 557511/2	56	1980	2012	8	1300	23.1	1145	575	18.8	652	298	9.6	338
Norway spruce	Germany GOE	ID S10511/1	70	1963	2011	10	1002	24.0	938	414	10.2	507	214	5.2	263
Norway spruce	Germany GOE	ID S10511/3	70	1963	2011	10	1961	20.6	1437	605	15.0	642	315	7.7	334
Norway spruce	Germany GOE	ID S20511/1	79	1967	2012	7	648	32.3	978	588	11.0	856	301	5.5	440
Norway spruce	Germany GOE	ID S20511/2	79	1967	2012	7	790	30.9	1110	717	22.4	984	368	11.3	506
Norway spruce	Germany GOE	ID S20511/3	79	1967	2012	7	628	35.1	1083	736	19.0	956	376	9.6	490
Norway spruce	Germany GOE	ID S22511/1	85	1967	2012	6	1152	27.3	1327	734	16.0	899	379	8.1	464
Norway spruce	Germany GOE	ID S22511/2	85	1967	2012	7	1088	27.3	1253	665	12.4	842	343	6.3	435
Norway spruce	Germany GOE	ID S24511/1	86	1967	2012	6	665	33.6	1069	760	12.6	1193	389	6.3	612
Norway spruce	Germany GOE	ID S26511/2	72	1963	2013	11	480	35.2	831	613	19.0	965	313	9.6	495
Norway spruce	Germany GOE	ID S26511/3	62	1963	2003	9	521	32.5	794	473	15.5	732	242	7.8	377
Norway spruce	Germany GOE	ID S26511/4	72	1963	2013	11	886	28.9	1118	738	17.2	962	380	8.7	496
Norway spruce	Germany MUE	111/5	97	1954	1992	6	286	44.6	724	771	12.4	1276	391	6.2	648
Norway spruce	Germany MUE	43832	122	1882	1972	15	492	44.7	1250	1364	15.6	2189	691	7.8	1115
Norway spruce	Germany MUE	43863	107	1882	1957	13	472	44.4	1187	1237	19.5	2063	627	9.6	1052
Norway spruce	Germany MUE	227/6	112	1959	2015	10	440	34.1	724	497	11.1	718	254	5.6	369

Norway spruce	Germany MUE	43864	116	1882	1965	14	480	45.2	1242	1338	15.9	1951	678	7.9	994
Norway spruce	Germany MUE	43835	143	1882	1990	18	496	47.6	1394	1505	16.6	2147	761	8.2	1091
Norway spruce	Germany MUE	43866	143	1882	1990	18	388	54.0	1335	1597	17.0	2290	804	8.4	1159
Norway spruce	Germany MUE	67/1	131	1902	1990	14	443	50.7	1378	1637	18.6	2377	826	9.2	1205
Norway spruce	Germany MUE	67/2	131	1902	1990	14	344	54.4	1198	1453	18.2	2460	731	9.0	1246
Norway spruce	Germany MUE	68/1	130	1902	1990	14	544	45.3	1412	1566	17.8	2271	793	8.9	1155
Norway spruce	Germany MUE	68/2	130	1902	1990	14	376	50.3	1155	1365	16.4	2300	689	8.1	1167
Norway spruce	Germany MUE	72/1	120	1906	1990	14	600	37.5	1150	912	10.1	1488	465	5.0	764
Norway spruce	Germany MUE	72/2	120	1906	1990	13	556	41.0	1230	1200	18.3	1735	610	9.2	886
Norway spruce	Germany MUE	73/1	119	1906	1983	12	672	36.9	1255	1123	16.4	1489	573	8.3	762
Norway spruce	Germany MUE	73/2	119	1906	1983	12	520	39.9	1101	1064	17.8	1606	541	8.9	821
Norway spruce	Germany MUE	43838	113	1882	1963	14	632	40.6	1376	1473	14.6	2214	749	7.3	1130
Norway spruce	Germany MUE	43869	119	1882	1969	15	476	45.3	1236	1425	14.0	2311	722	7.0	1177
Norway spruce	Germany MUE	84/2	127	1921	2007	13	472	46.8	1291	1331	14.9	2166	673	7.4	1101
Norway spruce	Sweden	682/8	64	1967	2007	7	1861	17.3	1029	511	17.5	604	268	9.0	317
Norway spruce	Sweden	901/7	56	1979	2006	5	1030	26.4	1122	663	21.6	693	342	11.0	358
Norway spruce	Sweden	905/2	81	1968	2007	8	890	27.4	1031	797	22.7	946	411	11.6	488
Norway spruce	Sweden	905/5	84	1968	2007	8	420	32.7	645	490	14.4	726	251	7.3	374
Norway spruce	Sweden	907/5	63	1971	2007	7	1520	19.8	1044	580	15.2	682	302	7.8	356
Norway spruce	Sweden	914/2	57	1975	2002	5	870	24.0	814	459	19.8	678	238	10.1	351
Norway spruce	Sweden	915/1	61	1968	2000	6	1590	20.2	1130	585	13.4	672	305	6.9	351
Norway spruce	Sweden	916/4	57	1968	1999	6	1630	20.3	1169	662	15.6	752	345	8.0	392
Norway spruce	Sweden	917/1	68	1972	2006	6	1770	16.3	888	388	14.6	526	204	7.6	276
Norway spruce	Sweden	920/9	71	1967	2001	7	1300	25.2	1313	846	18.3	1032	438	9.3	534
Norway spruce	Sweden	921/2	71	1966	2007	7	1070	25.9	1134	712	16.9	1044	368	8.5	541
Norway spruce	Sweden	925/3	62	1973	1999	5	1824	17.9	1070	486	9.5	530	254	4.9	277
Norway spruce	Sweden	928/5	51	1979	2007	6	940	22.0	768	413	19.2	680	214	9.9	354
Norway spruce	Sweden	928/7	51	1979	2007	6	1280	19.4	851	437	18.4	643	228	9.5	336
Norway spruce	Sweden	932/1	69	1968	2002	6	1940	17.0	1049	464	9.6	512	243	5.0	268

Norway spruce	Sweden	937/4	61	1974	2007	6	1310	24.8	1292	889	24.9	1101	460	12.7	570
Norway spruce	Sweden	941/7	64	1968	2001	7	1676	21.0	1265	690	25.1	839	359	12.8	438
Norway spruce	Sweden	943/6	72	1969	2003	7	590	26.5	647	379	17.9	612	196	9.1	317
Norway spruce	Sweden	943/9	72	1969	2003	7	1752	17.9	1028	487	17.5	579	255	9.0	303
Norway spruce	Sweden	944/2	66	1971	2006	6	1230	24.8	1215	762	25.8	903	394	13.1	468
Norway spruce	Sweden	950/3	68	1975	2015	7	1670	23.8	1547	1003	27.1	1133	519	13.9	587
Norway spruce	Sweden	950/4	68	1975	2015	7	830	29.0	1052	750	22.5	961	386	11.5	496
Norway spruce	Sweden	995/5	53	1979	2002	5	1080	25.0	1083	633	19.7	795	327	10.1	412
Norway spruce	Switzerland	1021000/1	149	1912	1993	15	462	51.8	1488	669	11.6	1211	337	5.8	615
Norway spruce	Switzerland	10260/1	65	1925	1954	6	600	26.6	662	500	15.0	831	258	7.5	430
Norway spruce	Switzerland	10440/1	82	1929	1963	7	425	35.2	734	613	17.3	1103	313	8.6	566
Norway spruce	Switzerland	21019000/1	94	1892	1963	13	624	38.4	1243	1062	16.9	1452	541	8.4	743
Norway spruce	Switzerland	21221000/1	96	1898	1975	12	693	32.0	1027	572	9.0	927	293	4.5	477
Norway spruce	Switzerland	21222000/1	85	1910	1969	10	1204	26.2	1299	542	9.9	877	280	5.0	454
Norway spruce	Switzerland	21275000/1	71	1905	1945	8	1626	21.7	1295	433	12.6	712	225	6.5	371
Norway spruce	Switzerland	212760/1	94	1908	1947	9	237	41.7	539	471	4.6	843	239	2.3	429
Norway spruce	Switzerland	212760/2	89	1908	1942	8	293	38.8	593	492	13.4	870	251	6.7	444
Norway spruce	Switzerland	21290000/1	109	1918	1981	12	338	35.7	597	430	10.8	774	220	5.5	398
Norway spruce	Switzerland	4011000/1	72	1937	1974	8	412	37.5	790	625	20.5	1020	318	10.3	522
Norway spruce	Switzerland	4012000/1	73	1937	1974	8	424	35.1	731	558	21.2	1000	285	10.6	514
